# Association of cardiometabolic microRNAs with COVID-19 severity and mortality

**DOI:** 10.1093/cvr/cvab338

**Published:** 2021-11-10

**Authors:** Clemens Gutmann, Kseniya Khamina, Konstantinos Theofilatos, Andreas B Diendorfer, Sean A Burnap, Adam Nabeebaccus, Matthew Fish, Mark J W McPhail, Kevin O'Gallagher, Lukas E Schmidt, Christian Cassel, Georg Auzinger, Salvatore Napoli, Salma F Mujib, Francesca Trovato, Barnaby Sanderson, Blair Merrick, Roman Roy, Jonathan D Edgeworth, Ajay M Shah, Adrian C Hayday, Ludwig Traby, Matthias Hackl, Sabine Eichinger, Manu Shankar-Hari, Manuel Mayr

**Affiliations:** 1 King's College London British Heart Foundation Centre, School of Cardiovascular Medicine and Sciences, 125 Coldharbour Lane, London SE5 9NU, UK; 2 TAmiRNA GmbH, Leberstrasse 20, Vienna 1110, Austria; 3 King's College Hospital NHS Foundation Trust, Denmark Hill, London SE5 9RS, UK; 4 Peter Gorer Department of Immunobiology, School of Immunology and Microbial Sciences, King's College London, Great Maze Pond, London, SE1 9RT, UK; 5 Department of Intensive Care Medicine, Guy's and St Thomas' NHS Foundation Trust, Westminster Bridge Road, London SE1 7EH, UK; 6 Department of Inflammation Biology, School of Immunology and Microbial Sciences, Faculty of Life Sciences and Medicine, King's College London, Newcomen Street, London SE1 1UL, UK; 7 Institute of Liver Studies, King's College Hospital, Denmark Hill, London SE5 9RS, UK; 8 Department of Liver Intensive Care & Critical Care, King's College Hospital London, Denmark Hill, London SE5 9RS, UK; 9 Department of Critical Care, Cleveland Clinic London, 33 Grosvenor Place, London SW1X 7HY, UK; 10 Centre for Clinical Infection and Diagnostics Research, Department of Infectious Diseases, Guy’s and St Thomas’ NHS Foundation Trust & King’s College London, Westminster Bridge Road, London SE1 7EH, UK; 11 The Francis Crick Institute, 1 Midland Road, London NW1 1AT, UK; 12 Department of Medicine I, Division of Infectious Diseases and Tropical Medicine, Medical University of Vienna, Waehringer Guertel 18-20, 1090 Vienna, Austria; 13 Department of Medicine I, Division of Haematology and Hemostaseology Medical University of Vienna, Waehringer Guertel 18-20, 1090 Vienna, Austria; 14 Centre of Inflammation Research, The University of Edinburgh, 47 Little France Crescent, Edinburgh EH16 4TJ, UK

**Keywords:** COVID-19, SARS-CoV-2, MicroRNAs, RNA-Seq, Proteomics, Biomarkers

## Abstract

**Aims:**

Coronavirus disease 2019 (COVID-19) can lead to multiorgan damage. MicroRNAs (miRNAs) in blood reflect cell activation and tissue injury. We aimed to determine the association of circulating miRNAs with COVID-19 severity and 28 day intensive care unit (ICU) mortality.

**Methods and results:**

We performed RNA-Seq in plasma of healthy controls (*n *=* *11), non-severe (*n *=* *18), and severe (*n *=* *18) COVID-19 patients and selected 14 miRNAs according to cell- and tissue origin for measurement by reverse transcription quantitative polymerase chain reaction (RT–qPCR) in a separate cohort of mild (*n *=* *6), moderate (*n *=* *39), and severe (*n *=* *16) patients. Candidates were then measured by RT–qPCR in longitudinal samples of ICU COVID-19 patients (*n *=* *240 samples from *n *=* *65 patients). A total of 60 miRNAs, including platelet-, endothelial-, hepatocyte-, and cardiomyocyte-derived miRNAs, were differentially expressed depending on severity, with increased miR-133a and reduced miR-122 also being associated with 28 day mortality. We leveraged mass spectrometry-based proteomics data for corresponding protein trajectories. Myocyte-derived (myomiR) miR-133a was inversely associated with neutrophil counts and positively with proteins related to neutrophil degranulation, such as myeloperoxidase. In contrast, levels of hepatocyte-derived miR-122 correlated to liver parameters and to liver-derived positive (inverse association) and negative acute phase proteins (positive association). Finally, we compared miRNAs to established markers of COVID-19 severity and outcome, i.e. SARS-CoV-2 RNAemia, age, BMI, D-dimer, and troponin. Whilst RNAemia, age and troponin were better predictors of mortality, miR-133a and miR-122 showed superior classification performance for severity. In binary and triplet combinations, miRNAs improved classification performance of established markers for severity and mortality.

**Conclusion:**

Circulating miRNAs of different tissue origin, including several known cardiometabolic biomarkers, rise with COVID-19 severity. MyomiR miR-133a and liver-derived miR-122 also relate to 28 day mortality. MiR-133a reflects inflammation-induced myocyte damage, whilst miR-122 reflects the hepatic acute phase response.

## Graphical Abstract

**Figure:**
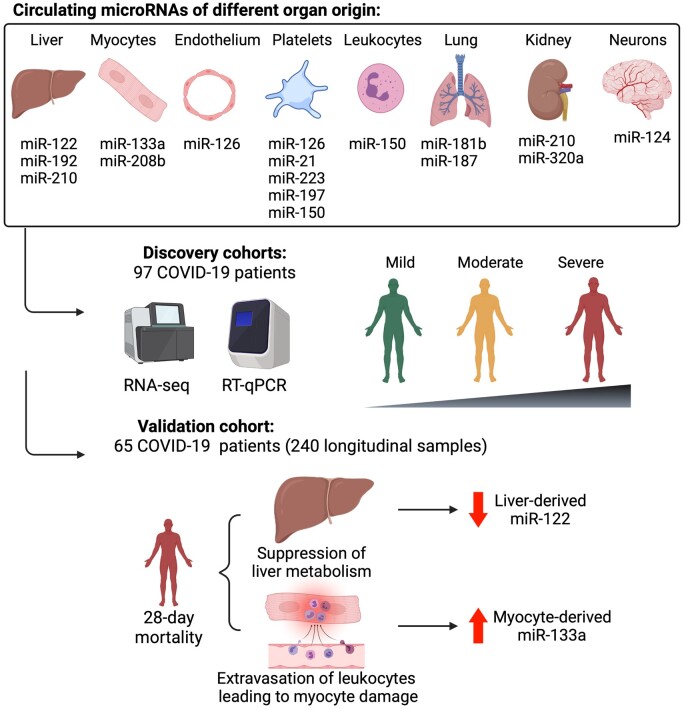

**Time for primary review: 29 days.**

## 1. Introduction

Coronavirus disease 2019 (COVID-19), caused by the single-stranded RNA virus SARS-CoV-2, varies in severity from mild self-limiting disease to critical illness with multiorgan failure. Cardiovascular involvement in severe COVID-19 ranges from elevated cardiac damage markers, venous and arterial thrombosis to arrhythmias, and myocardial infarctions.^1^ Similarly, liver dysfunction is prevalent in COVID-19 patients^2^ and cardiometabolic risk factors are associated with COVID-19 severity and mortality.^3^ Therefore, analysing markers of organ damage might inform on adverse outcomes and COVID-19 complications.

MicroRNAs (miRNAs) are small non-coding RNAs (∼22 nt in length) that repress synthesis of target proteins. miRNAs are also stably detectable in the circulation. Given their cell- and tissue-specific enrichment,^4^^,^^5^ miRNAs have been investigated as diagnostic and prognostic biomarkers for pathologies including myocardial infarction, liver failure, and sepsis,^5–9^ as well as drug-induced organ injury.^10^ Whilst protein biomarkers for COVID-19 have been extensively investigated,^11–15^ only two studies measured preselected circulating miRNAs in COVID-19 patients thus far.^16–18^

In this study, we performed next-generation sequencing (NGS) of small RNAs in healthy control individuals (*n *=* *11), non-severe (*n *=* *18), and severe (*n *=* *18) COVID-19 patients. miRNAs with a defined cell- and tissue origin (Supplementary material online, *Table S1*) were then validated by reverse transcription quantitative polymerase chain reaction (RT–qPCR) in an independent cohort of COVID-19 patients with different disease severities (*n *=* *61). Finally, validation was performed by longitudinal measurements in COVID-19 patients admitted to the intensive care unit (ICU, *n *=* *240 samples from *n *=* *65 patients). Circulating miRNAs were related to clinical parameters, 28 day ICU mortality, SARS-CoV-2 RNAemia, and proteomics by data-independent acquisition mass spectrometry (DIA-MS).^12^

## 2. Methods

### 2.1 Clinical data

####  

For small RNA-Seq of plasma samples, we obtained blood from COVID-19 patients with mild to moderate disease (i.e. WHO ordinal scale ≤5, *n *=* *18) and severe disease (i.e. WHO ordinal scale >5, *n *=* *18) upon hospitalization at the Vienna General Hospital, Austria. In addition, blood from healthy controls (*n *=* *11) was collected. For RT–qPCR of plasma samples, we obtained blood from COVID-19 ward patients with mild (i.e. WHO ordinal scale 1–2, *n *=* *6) and moderate disease (i.e. WHO ordinal scale 3–5, *n *=* *39) upon hospitalization at St Thomas’ Hospital, London, UK. In addition, we obtained plasma samples from patients with severe disease (i.e. WHO ordinal scale >5, *n *=* *16) within 6 days of admission to the ICU of King’s College Hospital, London, UK. Plasma was collected in EDTA BD Vacutainer™ tubes (BD, 362799) and centrifuged at 2000× *g* for 15 min. Moreover, we obtained longitudinal serum samples from severe COVID-19 ICU patients (*n *=* *240 samples from *n *=* *65 patients) recruited at St Thomas’ Hospital and at King’s College Hospital. Serum from King’s College Hospital ICU patients originated from the same blood donation as the plasma King’s College Hospital ICU samples. Serum was collected in silica BD Vacutainer™ tubes (BD, 367820) and left to clot for 15 min, followed by centrifugation at 2000× *g* for 15 min. Serum samples were collected within 6 days of admission to ICU and thereafter during Weeks 2 and 3 of ICU admission. Recruitment of samples used for RNA-Seq occurred between August 2018 and January 2020 (healthy volunteers) and between May and December 2020 (COVID-19 patients). Recruitment of samples used for RT–qPCR occurred between March 2020 and July 2020. The study was conducted in accordance with the Declaration of Helsinki and approved by an institutional review board (EK1404/2020 for COVID-19 patients and EK112/2010 for healthy volunteers recruited at the Vienna General Hospital; REC19/NW/0750 for patients recruited at King’s College Hospital; REC19/SC/0187 for severe ICU patients recruited at St Thomas’ Hospital; REC19/SC/0232 for mild and moderate patients recruited at St Thomas’ Hospital). Written informed consent was obtained directly from patients (if mentally competent), or from the next of kin or professional consultee. The consent procedure was then completed with retrospective consent if the patient regained capacity. The 28 day mortality was used as the primary outcome measure. Baseline clinical characteristics and demographics of our cohorts are shown in Supplementary material online, *Tables S2, S5**,**and S8*.^12^

### 2.2 RNA extraction for small RNA-Seq

Total RNA was extracted from 200 μL of citrate/CTAD plasma using the Maxwell RSC miRNA Tissue kit (Promega, AS1460) according to the manufacturer’s protocol. Briefly, samples were thawed on ice and centrifuged at 12 000× *g* for 5 min to remove cellular debris. For each sample, 200 μL of plasma were mixed with the following reagents: 200 μL of homogenization solution, 200 μL of lysis master mix containing 1 μL of pre-diluted 1:250 RNA spike-in mix (UniSp 2, 4, 5, Qiagen, 339390), and 15 μL of Proteinase K. Next, samples were incubated for 15 min on a heat block at 37°C, 300 rpm. After the incubation, samples were transferred to RSC cartridges followed by automated RNA extraction in the Maxwell instrument. Finally, total RNA was eluted in 50 μL nuclease-free water and stored at −80°C prior to further analyses.

### 2.3 Library preparation for small RNA-Seq

Library preparation was performed using RealSeq-Biofluids Plasma/Serum miRNA Library kit for Illumina sequencing (RealSeq Biosciences, 600-00048; protocol 20181220_RealSeq-BF_CL) according to the manufacturer’s protocol. Briefly, 8.5 μL of extracted RNA were used as input. The adapters were pre-diluted 1:4 to account for low miRNA abundance. Adapter-ligated libraries were circularized, reverse transcribed, and amplified. Library PCR was performed using 21 cycles with Illumina primers included in the kit. In total, 47 miRNA libraries were prepared from plasma samples and analysed for library fragments distribution using the Agilent DNA 1000 kit (Agilent Technologies, 5067-1504) with Agilent DNA1000 reagents (Agilent Technologies, 5067-1505).

The generated libraries were pooled in an equimolar proportion and the obtained pool was size-selected with the BluePippin system using a 3% agarose cassette, 100–250 kb (Sage Science, BDQ3010) to remove DNA fragments outside of the target range. The pooled and purified libraries were analysed for fragment distribution on an Agilent High Sensitivity DNA kit (Agilent Technologies, 5067-4626) with Agilent High Sensitivity DNA reagents (Agilent Technologies, 5067-4627). The library pool was then sequenced on an Illumina NextSeq550 (single-read, 75 bp) according to the manufacturer’s protocol at the Vienna BioCenter Core Facilities, Vienna, Austria. The RNA-Seq data have been deposited to the GEO NCBI repository with the accession number GSE176498.

### 2.4 RNA isolation and heparinase treatment

The miRNeasy Mini kit (Qiagen, 217004) was used according to the manufacturer’s recommendations for isolation of total RNA. For normalization of miRNAs, an exogenous miRNA (*Cel-miR-39-3p*, Qiagen, 219600) was spiked in during the first step of RNA isolation as described previously.^5^ Elution of RNA was performed in 30 μL of nuclease-free H_2_O by centrifugation at 8500× *g* for 1 min at 4°C. RNA was then treated with heparinase to overcome the confounding effect of heparin on qPCR,^5^^,^^19^ as described previously.^5^^,^^12^^,^^19^ Briefly, 8 μL of RNA was added to 2 μL of heparinase 1 from Flavobacterium heparinum (Sigma, H2519), 0.4 μL RNase inhibitor (RiboLock 40 U/μL, ThermoFisher, EO0381), 5.6 μL of heparinase buffer (pH 7.5) and incubated at 25°C for 3 h.^5^^,^^12^^,^^19^

### 2.5 Reverse transcription quantitative polymerase chain reaction

For detection of SARS-CoV-2 RNA, we performed a two-step RT–qPCR using the LunaScript^®^ RT SuperMix Kit (NEB, E3010) and the Luna Universal Probe qPCR Master Mix (NEB, M3004), as described previously.^12^ Samples were considered positive for SARS-CoV-2 if the cycle quantification (Cq) value of either N1 or N2 primer was below 40.

For miRNAs, the miRCURY LNA RT kit (Exiqon, 339340) was used for RT, whilst the miRCURY SYBR Green qPCR (Exiqon, 339347) in combination with miRCURY LNA miRNA PCR Assays (both Qiagen, Supplementary material online, *Table S1*) were used for qPCR detection, as described previously.^5^ Reactions were loaded using a Bravo Automated Liquid Handling Platform (Agilent). qPCR was performed on a ViiA7 Real-Time PCR System (Applied Biosystems) at 95°C for 2 min followed by 40 cycles of 95°C for 10 s and 56°C for 1 min. Relative quantification (RQ) of miRNAs was based on the 2^−ΔΔCq^ method,^4^ using *Cel-miR-39-3p* for ΔCq and a calibrator sample consisting of two identical replicates of equal volumes from all samples for ΔΔCq. RQ was performed using Microsoft Excel, version 15.32 for MacOS.

### 2.6 DIA-MS analysis

As described before,^12^ serum destined for DIA-MS analysis was inactivated using 1% (v/v) Triton X-100 and 1% (v/v) tributyl phosphate. Inactivated serum was denatured, reduced, and alkylated before enzymatic digestion with Trypsin/LysC (Promega, V5072). Digested peptide solutions were acidified and cleaned up using a Bravo AssayMAP Liquid Handling Platform equipped with C18 Cartridges (Agilent). PQ500 Reference Peptides (Biognosys, Ki-3019-96) were added to the cleaned serum peptide solutions. Peptides were analysed using a capillary flow reversed-phase liquid chromatography (LC)-MS system consisting of an UltiMate 3000 LC system (Thermo Scientific) and an Orbitrap Fusion Lumos Tribrid mass spectrometer (Thermo Scientific). Higher-energy C-trap dissociation was used to sequentially fragment precursors grouped into 30 isolation windows. Orbitrap MS1 and MS2 spectra were analysed using the PQ500 analysis plug-in provided in Spectronaut 14 (Biognosys). Quality control of peptide identifications was achieved by introducing *Q*-value, signal-to-noise ratio, and missing value thresholds. Quantification was based on MS2 peak area and target-to-reference ratio, i.e. the abundance ratio of a given serum peptide and its corresponding PQ500 reference peptide. Protein abundances were calculated by adding up quality-controlled peptide abundances. The mass spectrometry proteomics data have been deposited to the ProteomeXchange Consortium via the PRIDE partner repository with the dataset identifier PXD024089.^12^

### 2.7 Statistical analysis

#### 2.7.1 RNA-Seq

Analysis of RNA-Seq data was performed with the software package MiND, a data analysis pipeline that generates overall QC data, unsupervised clustering analysis, normalized miRNA count matrices, and differential expression analysis based on raw NGS data. Overall quality of the NGS data was evaluated automatically and manually with fastQC v0.11.8^20^ and multiQC v1.7.^21^ Reads from all passing samples were adapter trimmed and quality filtered (min Phred score of 30) using cutadapt v2.3^22^ and filtered for a minimum length of 17 nt. Mapping steps were performed with bowtie v1.2.2^23^ and miRDeep2 v2.0.1.2,^24^ whereas reads were mapped first against the genomic reference GRCh38.p12 provided by Ensembl^25^ allowing for two mismatches and subsequently miRBase v22.1,^26^ filtered for miRNAs of *Homo sapiens* only, allowing for one mismatch. For a general RNA composition overview, non-miRNA mapped reads were mapped against RNAcentral^27^ and then assigned to various RNA species of interest. Statistical analysis of preprocessed NGS data was done with R v3.6 and the packages ‘corrplot’, ‘Hmisc’, and ‘genefilter v1.68’. Differential expression analysis with edgeR v3.28^28^ used the quasi-likelihood negative binomial generalized log-linear model functions provided by the package. The independent filtering method of DESeq2^29^ was adapted for use with edgeR to remove low abundant miRNAs and thus optimize the Benjamini–Hochberg false discovery rate (FDR) correction.

#### 2.7.2 RT–qPCR, proteomics, and clinical data

Missing values of variables were imputed if <30% of the values were missing using K nearest neighbours-based imputation with *K* = 5. Variables with ≥30% missing values were excluded or analysed as binary variables in the case of RT–qPCR measurements of miRNAs (i.e. detectable vs. undetectable). Normal distribution of data was assessed after logarithmic (log_2_) transformation using the Shapiro–Wilk test. Significance between two groups of continuous variables was assessed using Student’s *t*-tests if data were normally distributed and using Mann–Whitney *U* tests if data were not normally distributed. Significance between more than two groups of continuous variables was assessed using ANOVA if data were normally distributed and Kruskal–Wallis if data were not normally distributed. The χ^2^ test was used to compare binary variables. A *P*-value threshold of 0.05 was used to infer significant changes. Benjamini and Hochberg’s correction was used to correct for multiple testing. Corrections for age, sex, and body mass index (BMI) were applied using the limma package.^30^ Spearman correlation was used to determine correlations between continuous variables. Point-biserial correlation was used to determine correlations between continuous and binary variables. All statistical analyses were two-tailed. Longitudinal miRNA trajectories were fitted using generalized alternative models. Generalized linear mixed models (binomial family) were fitted to combine binary and triplet signatures for the classification of severity or 28 days outcome of COVID-19 ICU patients using the ‘glmer’ R package. Empirical receiver operating characteristic (ROC) plots were created to calculate the area under the curves (AUC) using five-fold cross validation and bootstrapping to better assess their generalization properties. Kaplan–Meier analyses were conducted using the ‘survminer’ R library. GraphPad Prism (version 9.1.1) and R programming environment (v3.6) were used for statistical analysis and to generate associated figures. Schematic diagrams were created with Biorender.com.

## 3. Results

### 3.1 NGS of small RNAs in patients with different COVID-19 severity and healthy controls

To assess circulating miRNA changes, libraries of small RNAs were generated from plasma of healthy controls (*n *=* *11) and patients with mild/moderate (*n *=* *18) and severe (*n *=* *18) COVID-19 (admitted to the Vienna General Hospital, Austria, Supplementary material online, *Table S2*). Patients with non-severe and severe COVID-19 had similar comorbidities and did not differ in age and sex. Patients with severe COVID-19 had a marginally higher BMI [26.8 IQR (24.3, 30.8) vs. 30.1 IQR (27.7, 36.7), *P *=* *0.07, Supplementary material online, *Table S2*].

**Figure 1:**
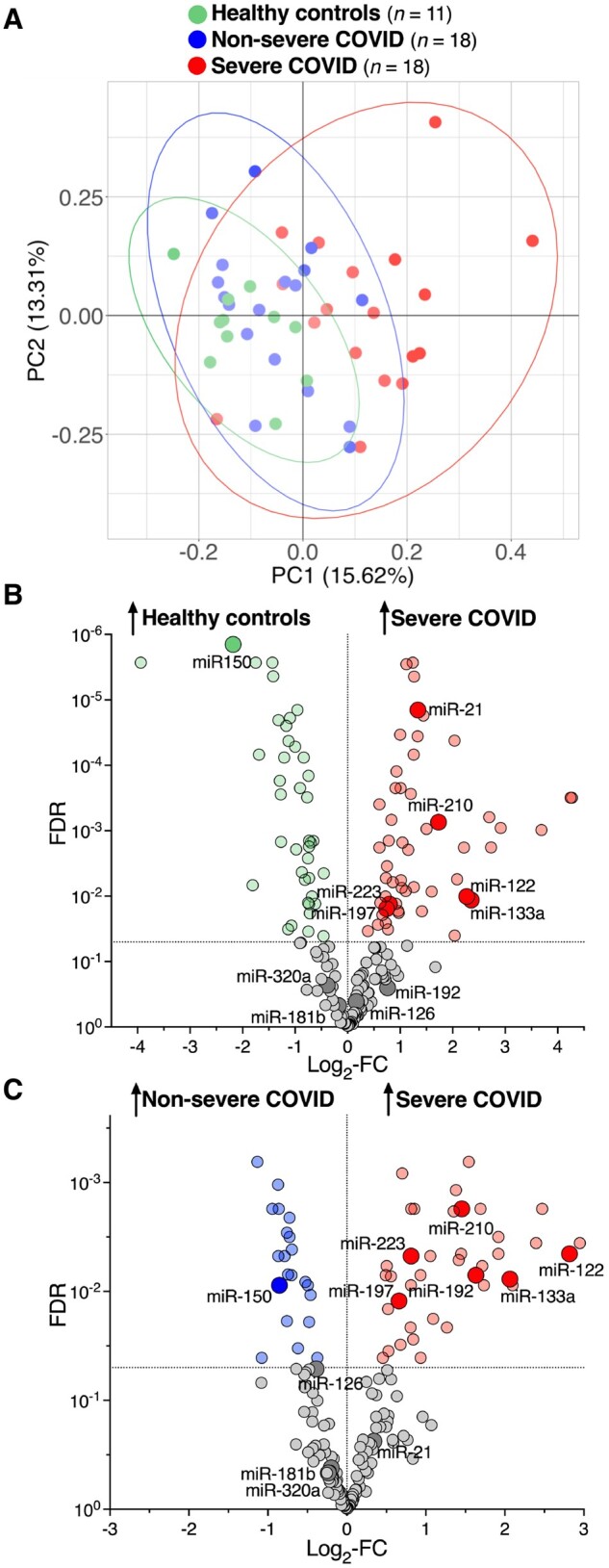
NGS of small RNAs in patients with different COVID-19 patients with different disease severity and healthy controls. (*A*) PC analysis based on RNA-Seq in plasma of healthy controls (*n *=* *11), non-severe (*n *=* *18), and severe (*n *=* *18) COVID-19 patients. PC1 (*x*-axis) and PC2 (*y*-axis) explain 15.62% and 13.31% of the variance, respectively. (*B*) Volcano plot showing plasma miRNAs that are differentially expressed between healthy controls (*n *=* *11) and severe (*n *=* *18) COVID-19 patients. (*C*) Volcano plot showing plasma miRNAs that are differentially expressed between non-severe (*n *=* *18) and severe (*n *=* *18) COVID-19 patients. Highlighted are miRNAs that have previously been attributed a role in critically ill patients or are miRNAs with a tissue-specific origin (Supplementary material online, *Table S1*). Differential expression analysis of RNA-Seq data was performed using edgeR and applying the independent filtering method of DESeq2 to remove low abundant miRNA to optimize the Benjamini–Hochberg FDR correction. All statistical analyses are two-tailed.

Without distinguishing between isomiRs, 333 miRNAs were consistently identified in all samples, including miRNAs for which a role in critically ill patients or a tissue-specific origin has been shown previously (Supplementary material online, *Table S1*). Differences in the plasma miRNome of healthy controls (*n *=* *11), non-severe (*n *=* *18), and severe (*n *=* *18) COVID-19 were visualized by principal component (PC) analyses (*Figure 1A*). A total of 94 and 60 miRNAs differed significantly (FDR < 0.05) between healthy controls and patients with severe COVID-19 (*Figure 1B* and Supplementary material online, *Table S3*) and between patients with mild/moderate COVID-19 and severe COVID-19 (*Figure 1C* and Supplementary material online, *Table S4*), respectively. A total of 109 miRNAs were different (FDR < 0.05) between healthy controls and patients with mild/moderate COVID-19 (Supplementary material online, *Figure S1*). Two tissue-specific miRNAs, liver-derived miR-122,^6^ and myocyte-derived (myomiR) miR-133a,^5^^,^^9^^,^^31^ were among the most up-regulated miRNAs in severe COVID-19 and remained significant after adjusting for BMI (miR-122: adjusted *P *=* *0.0096, FDR = 0.036, miR-133a: adjusted *P *=* *0.013, FDR = 0.044, Supplementary material online, *Table S4*).

**Figure 2:**
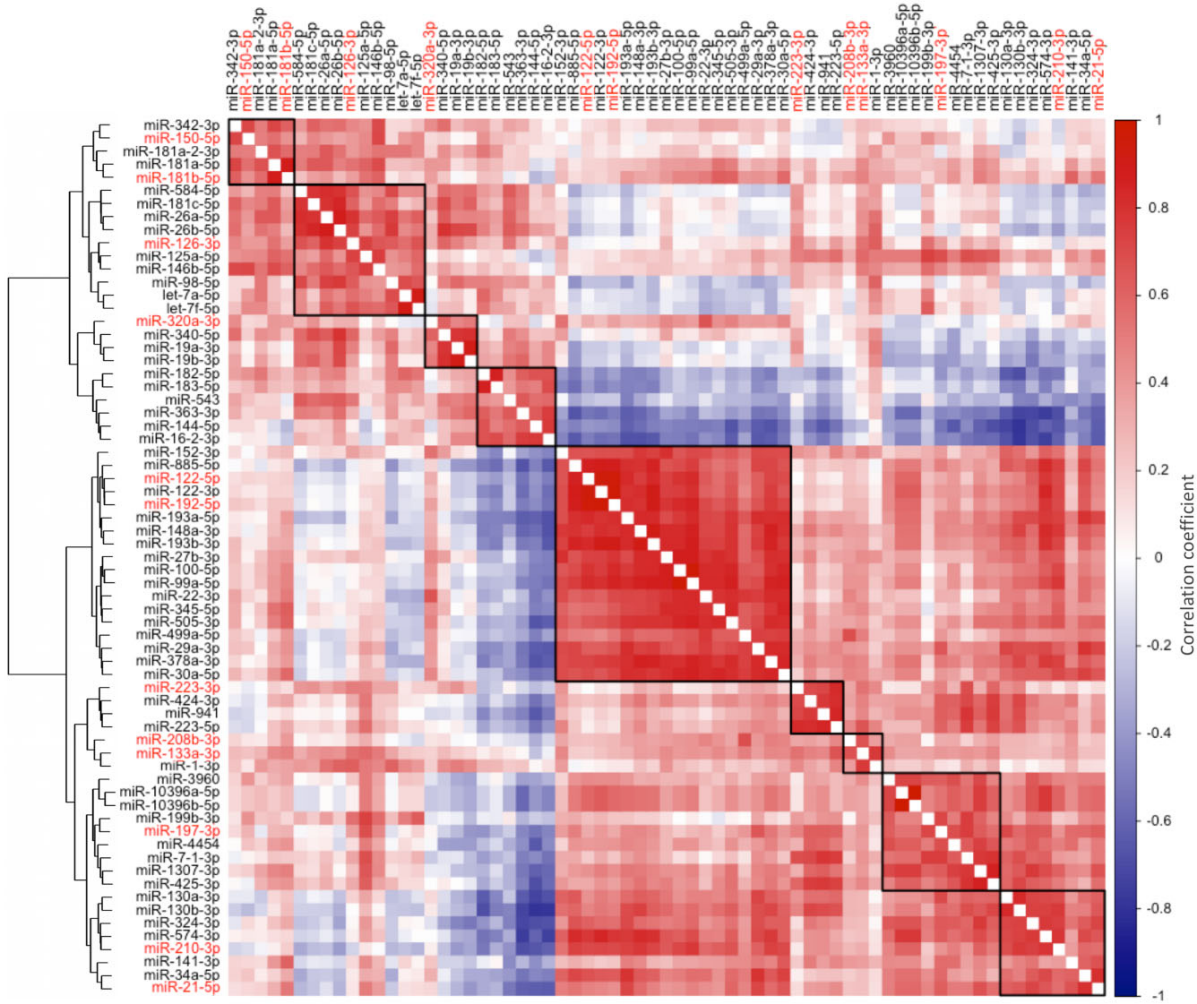
Clusters and correlations of circulating miRNAs measured by RNA-Seq in COVID-19 patients. The heat map represents a hierarchical cluster analysis conducted upon a Spearman correlation network of miRNA levels in COVID-19 patients (*n *=* *36) that were found to be differentially expressed between non-severe and severe COVID-19 patients and for which a role in critically ill patients or a tissue-specific origin has been shown previously (highlighted in red, Supplementary material online, *Table S1*).

We then performed a hierarchical clustering analysis on the miRNAs in COVID-19 patients that were found to be differentially expressed between non-severe and severe COVID-19 patients and for which a role in critically ill patients or a tissue-specific origin had been shown previously (*Figure 2*). As expected, miRNAs with a defined cellular origin clustered and correlated together. Liver-derived miR-122 clustered with and strongly correlated to other well-known liver-derived miRNAs, such as miR-192 (*r *=* *0.90, *P *<* *0.0001) and miR-885 (*r *=* *0.79, *P *<* *0.0001).^6^ Similarly, myocyte-derived miR-133a clustered with and strongly correlated to other well-known myomiRs, i.e. miR-1 (*r *=* *0.71, *P *<* *0.0001) and miR-208b (*r *=* *0.59, *P *<* *0.0001).^5^

### 3.2 RT–qPCR validation of miRNAs in COVID-19 patients with different disease severity

**Figure 3:**
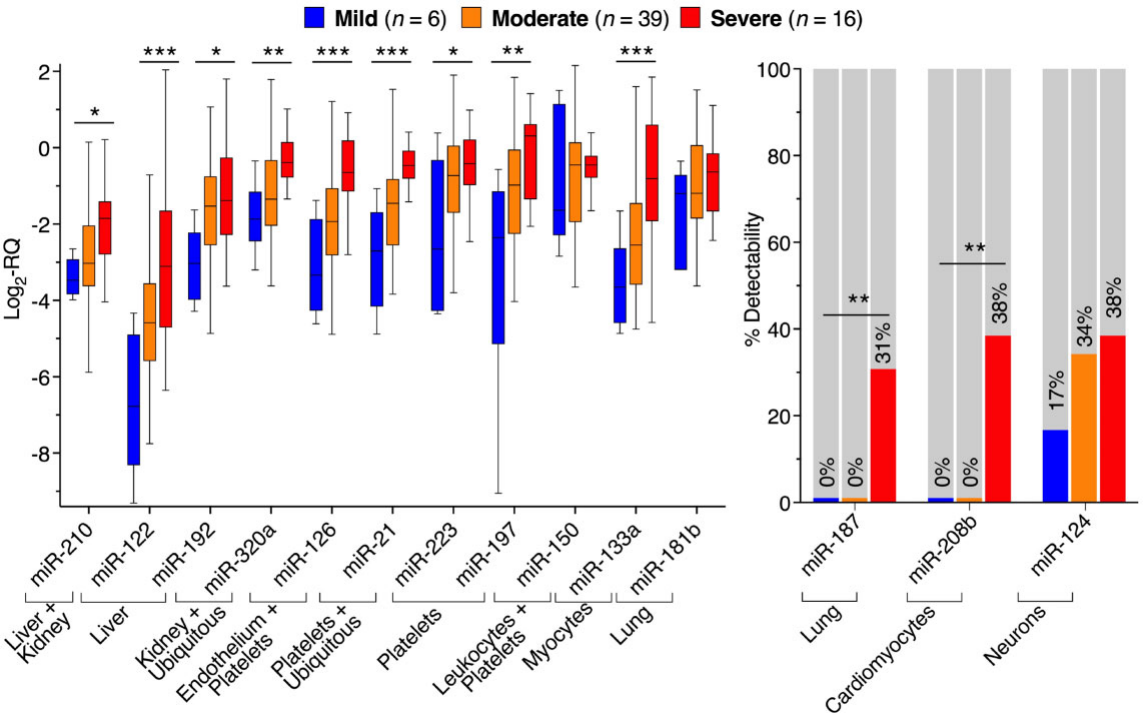
RT–qPCR validation of miRNAs in COVID-19 patients with different disease severity. RT–qPCR of miRNAs in plasma of mild (*n *=* *6), moderate (*n *=* *39), and severe (*n *=* *16) COVID-19 patients. Tukey boxplots depict the median (horizontal line), interquartile range (box borders), and 1.5× interquartile range (whiskers). Lung-derived miR-187, cardiomyocyte-derived miR-208b, and neuron-derived miR-124 had poor plasma RT–qPCR detectability and were therefore analysed as binary variables. Significance between the three severity groups was determined using ANOVA tests for continuous variables, χ^2^ tests for binary variables and then applying Benjamini and Hochberg’s correction for the 14 comparisons. *FDR <0.05. **FDR <0.01. ***FDR <0.001. A list of the FDR uncorrected and corrected for age, sex, and BMI is presented in Supplementary material online, *Tables S6 and S7*. All statistical analyses are two-tailed.

In an independent cohort of patients with mild (*n *=* *6), moderate (*n *=* *39), and severe (*n *=* *16) COVID-19 (admitted to King’s College Hospital, London, UK, Supplementary material online, *Table S5*), we selected 14 plasma miRNAs with different cellular and tissue origin (Supplementary material online, *Table S1*) for validation by RT–qPCR. Of them, 11 miRNAs were detectable in >70% of samples (*Figure 3* and Supplementary material online, *Table S6*). Three tissue-specific miRNAs (miR-187, miR-208b, and miR-124; *Figure 3* and Supplementary material online, *Table S7*) showed poor detectability and were analysed as binary variables based on detectability. In line with the NGS results, most miRNAs with a defined tissue origin; including miRNAs previously implicated as markers of the cardiometabolic system, such as platelet-, endothelial-, myocyte-, and hepatocyte-derived miRNAs; rose with COVID-19 severity (*Figure 3* and Supplementary material online, *Tables S6 and S7*). Others, i.e. miR-150, showed pronounced changes by RNA-Seq in comparison to healthy controls, but were not significantly changing with COVID-19 severity by RT–qPCR. In agreement with the NGS results, liver-derived miR-122^6^ and myocyte-derived miR-133a^5^^,^^9^^,^^31^ showed the largest fold change (FC) in mild vs. severe disease (miR-122 log_2_-FC = 3.66, FDR = 0.0008; miR-133a log_2_-FC = 2.84, FDR = 0.0008; *Figure 3* and Supplementary material online, *Table S6*). After adjustment for age, sex, and BMI, miR-122 and miR-133a were the only miRNAs besides platelet- and endothelium-derived miR-126^4^^,^^32–34^ that remained significant and were reliably detectable by RT–qPCR. Unlike most miRNAs (Supplementary material online, *Figure S2*), miR-122 (*r *=* *0.96, *P *<* *0.0001; Supplementary material online, *Figure S2A*) and miR-133a (*r *=* *0.62, *P *<* *0.0001; Supplementary material online, *Figure S2C*) levels were also highly correlated in serum and plasma. Given their strong association with COVID-19 severity and their consistency in plasma and serum, we further assessed the associations of miR-122 and miR-133a with 28 day ICU mortality.

### 3.3 Association of miR-133a levels with proteomics data, clinical parameters, and outcome

**Figure 4:**
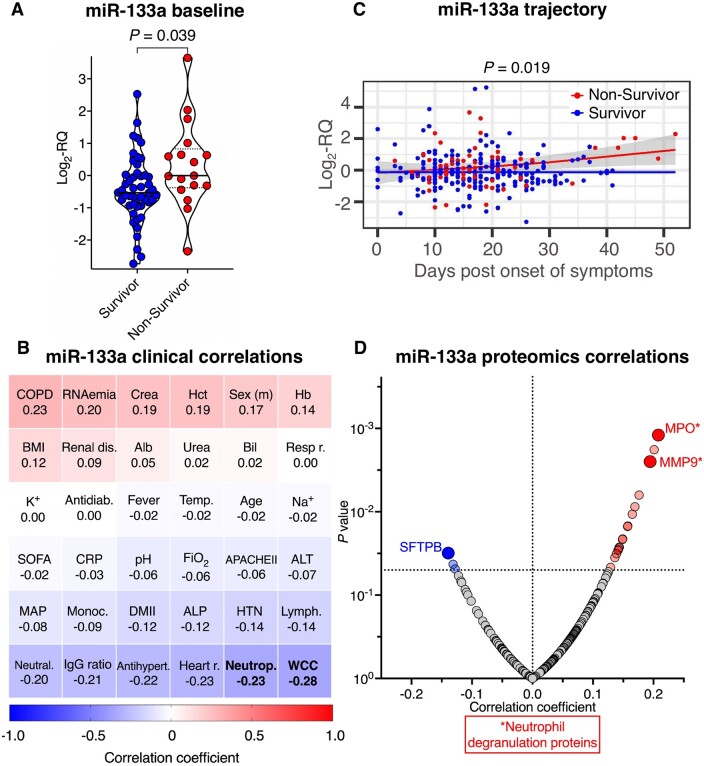
Association of miR-133a levels with proteomics data, clinical parameters and outcome. (*A*) Baseline miR-133a serum levels in COVID-19 ICU survivors (*n *=* *48) and non-survivors (*n *=* *17). Lines inside violin plots show median (continuous line) and interquartile range (dotted lines). A two-tailed, unpaired Student’s *t*-test was used to determine statistical significance. (*B*) Heatmap showing correlations of miR-133a levels with clinical characteristics of COVID-19 ICU patients (*n *=* *65) at baseline. Spearman correlation was used to determine correlations between continuous variables. Point-biserial correlation was used to determine correlations between continuous and binary variables. Significant (*P *<* *0.05) correlations are highlighted in bold font. (*C*) Trajectory of miR-133a in COVID-19 ICU survivors (*n *=* *48) and non-survivors (*n *=* *17) as a function of days post onset of symptoms. Lines show fitted generalized additive models with grey bands indicating the 95% CI, correcting for age, sex, and BMI. (*D*) Longitudinal protein correlations with miR-133a (*n *=* *240 samples from *n *=* *62 COVID-19 ICU patients). Significant (*P *<* *0.05) correlations are shown in blue (negative) and red (positive). Highlighted are neutrophil degranulation proteins and SFTPB. All statistical analyses are two-tailed. Alb, albumin; ALP, alkaline phosphatase; ALT, alanine aminotransferase; Antidiab, antidiabetic pre-medication; Antihypert, antihypertensive pre-medication; APACHEII, acute physiology and chronic health evaluation score; Bil, bilirubin; BMI, body mass index; COPD, chronic obstructive pulmonary disease; Crea, creatinine; CRP, C-reactive protein; DMII, type II diabetes mellitus; FiO_2_, fraction of inspired oxygen; Hb, haemoglobin; Hct, haematocrit; Heart r, heart rate; HTN, hypertension; IgG ratio, anti-SARS-CoV-2 IgG ratio measured by ELISA;^12^ K^+^, potassium; Lymph, lymphocytes; MAP, mean arterial pressure; Monoc, monocytes; MPO, myeloperoxidase; Na^+^, sodium; Neutral, anti-SARS-CoV-2 neutralization capacity measured by the surrogate virus neutralization test;^12^ Neutrop, neutrophils; MMP9, matrix metalloproteinase-9; Renal dis, renal disease; Resp r, respiratory rate; SFTPB, pulmonary surfactant-associated protein B; SOFA, sequential organ failure assessment score; Temp, body temperature; WCC, white cell count.

Serum from 65 COVID-19 patients was sampled within 6 days of ICU admission (St Thomas’ Hospital and King’s College Hospital, London, UK, Supplementary material online, *Table S8*) and thereafter during Weeks 2 and 3 of ICU admission. Baseline levels of myomiR miR-133a were higher in non-survivors (unadjusted *P *=* *0.039; age and sex-adjusted *P *=* *0.058; age, sex and BMI-adjusted *P *=* *0.0545; *Figure 4A*) and negatively correlated with neutrophil count (*r* = −0.27, *P *=* *0.040) and total white cell count (*r* = −0.28, *P *=* *0.035; *Figure 4B*). In longitudinal measurements as a function of days post onset of symptoms, miR-133a showed an increasing trajectory in non-survivors compared with survivors (age, sex, and BMI-adjusted *P *=* *0.019, *Figure 4C*). Correlating miR-133a to longitudinal DIA-MS proteomics data,^12^ myeloperoxidase (MPO) showed the strongest positive correlation to miR-133a (*r *=* *0.21, *P *=* *0.001, *Figure 4D* and Supplementary material online, *Table S9*) alongside other neutrophil degranulation proteins (i.e. matrix metalloproteinase-9), complement (mannan-binding lectin serine protease 2, complement component C8 alpha, C1-inhibitor, complement factor H), and coagulation proteins (thrombin, protein Z). The protein with the strongest negative association was pulmonary surfactant-associated protein B (SFTPB) (*r* = −0.14, *P *=* *0.03, *Figure 4D* and Supplementary material online, *Table S9*), a lung-derived serum protein.

### 3.4 Association of miR-122 levels with proteomics data, clinical parameters, and outcome

**Figure 5:**
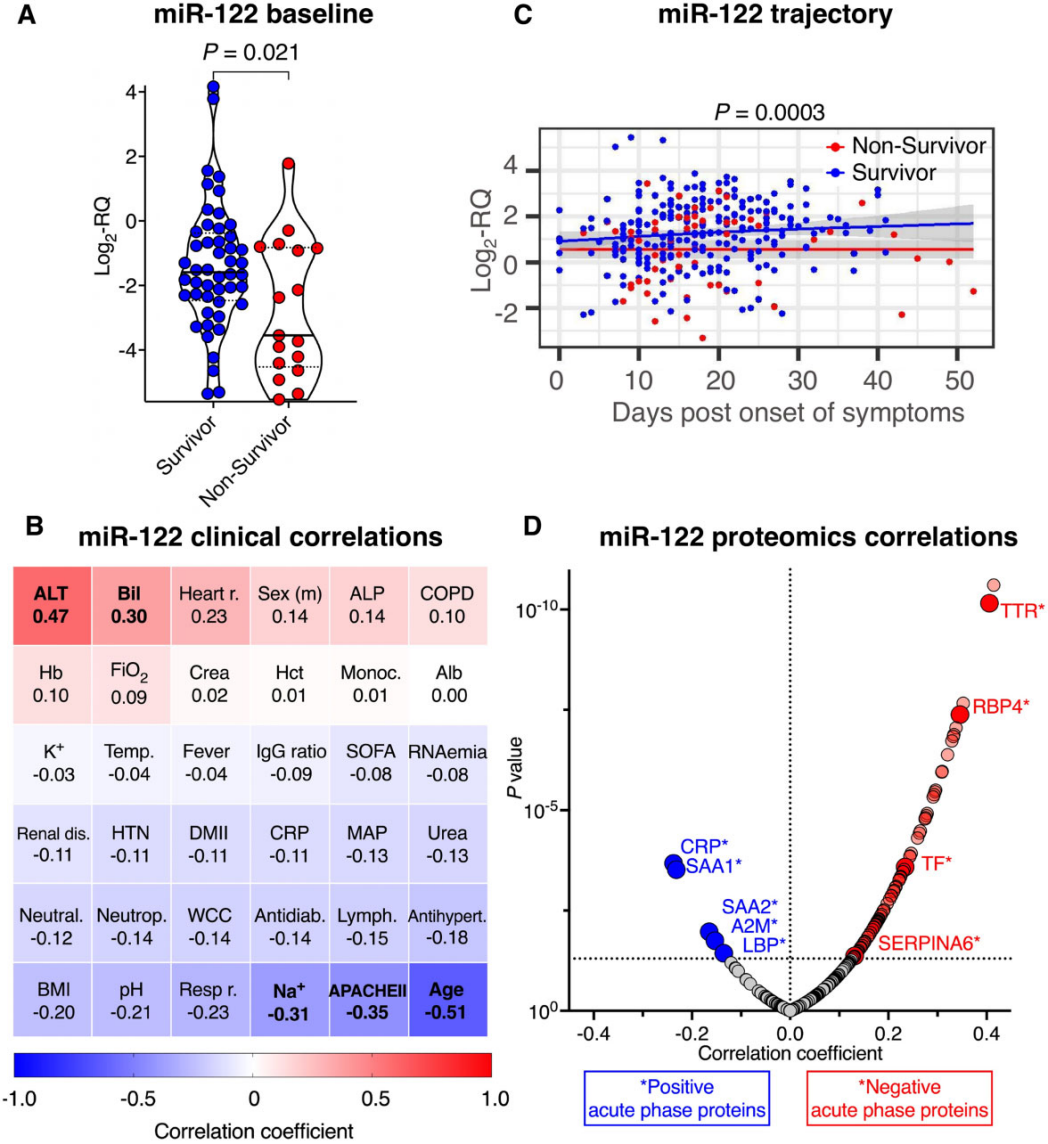
Association of miR-122 levels with proteomics data, clinical parameters, and outcome. (*A*) Baseline miR-122 levels in COVID-19 ICU survivors (*n *=* *48) and non-survivors (*n *=* *17). Lines inside violin plots show median (continuous line) and interquartile range (dotted lines). Two-tailed, unpaired Student’s *t*-test was used to determine statistical significance. (*B*) Heatmap showing correlations of miR-122 levels with clinical characteristics of COVID-19 ICU patients (*n *=* *65) at baseline. Spearman correlation was used to determine correlations between continuous variables. Point-biserial correlation was used to determine correlations between continuous and binary variables. Significant (*P *<* *0.05) correlations are highlighted in bold font. (*C*) Trajectory of miR-122 in COVID-19 ICU survivors (*n *=* *48) and non-survivors (*n *=* *17) as a function of days post onset of symptoms. Lines show fitted generalized additive models with grey bands indicating the 95% CI, correcting for age, sex, and BMI. (*D*) Longitudinal protein correlations with miR-122 (*n *=* *240 samples from *n *=* *62 COVID-19 ICU patients). Significant (*P *<* *0.05) correlations are highlighted in blue (negative) and red (positive). Highlighted are positive and negative APPs. All statistical analyses are two-tailed. A2M, alpha-2-macroglobulin; CRP, C-reactive protein; LBP, lipopolysaccharide-binding protein; RBP4, retinol-binding protein 4; SAA1, serum amyloid A-1 protein; SAA2, serum amyloid A-2 protein; SERPINA6, corticosteroid-binding globulin; TF, serotransferrin; TTR, transthyretin.

In contrast to myomiR miR-133a, hepatocyte-derived miR-122 baseline levels were significantly lower in non-survivors in an unadjusted analysis (*P *=* *0.021, *Figure 5A*). Due to a strong inverse correlation of miR-122 with age (*r* = −0.51, *P *<* *0.0001, *Figure 5B*), this association of baseline levels did not withstand adjustment for age, sex, and BMI (age and sex-adjusted *P *=* *0.69; age, sex, and BMI-adjusted *P *=* *0.65). Baseline levels of miR-122 correlated positively with alanine aminotransferase (*r *=* *0.47, *P *=* *0.0007), bilirubin (*r *=* *0.29, *P *=* *0.025) and inversely with sodium (*r* = −0.31, *P *=* *0.016) and the APACHE II score (*r* = −0.35, *P *=* *0.005, *Figure 5B*). In longitudinal measurements as a function of days post onset of symptoms, miR-122 was decreased in non-survivors and significance was retained after adjustment for age, sex, and BMI (adjusted *P *=* *0.0003, *Figure 5C*). Levels of miR-122 correlated positively with afamin, apolipoproteins, complement, and coagulation factors, including three proteins that we previously found to have an inverse association with 28 day mortality: fibronectin 1, plasminogen and Vitamin-K-dependent protein C (*Figure 5D* and Supplementary material online, *Table S10*).^12^ Circulating miR-122 was inversely related to hepatic acute phase proteins (APPs) that increase upon infections (positive APPs, i.e. alpha 2-macroglobulin, lipopolysaccharide (LPS)-binding protein, C-reactive protein, serum amyloid A-1, and serum amyloid A-2).^35^ Accordingly, circulating miR-122 levels were positively correlated to hepatic APPs that decrease upon infections (negative APPs, i.e. serotransferrin, transthyretin, retinol-binding protein 4 and corticosteroid-binding globulin).

### 3.5 Circulating miRNAs as part of COVID-19 severity and outcome classification signatures

**Figure 6:**
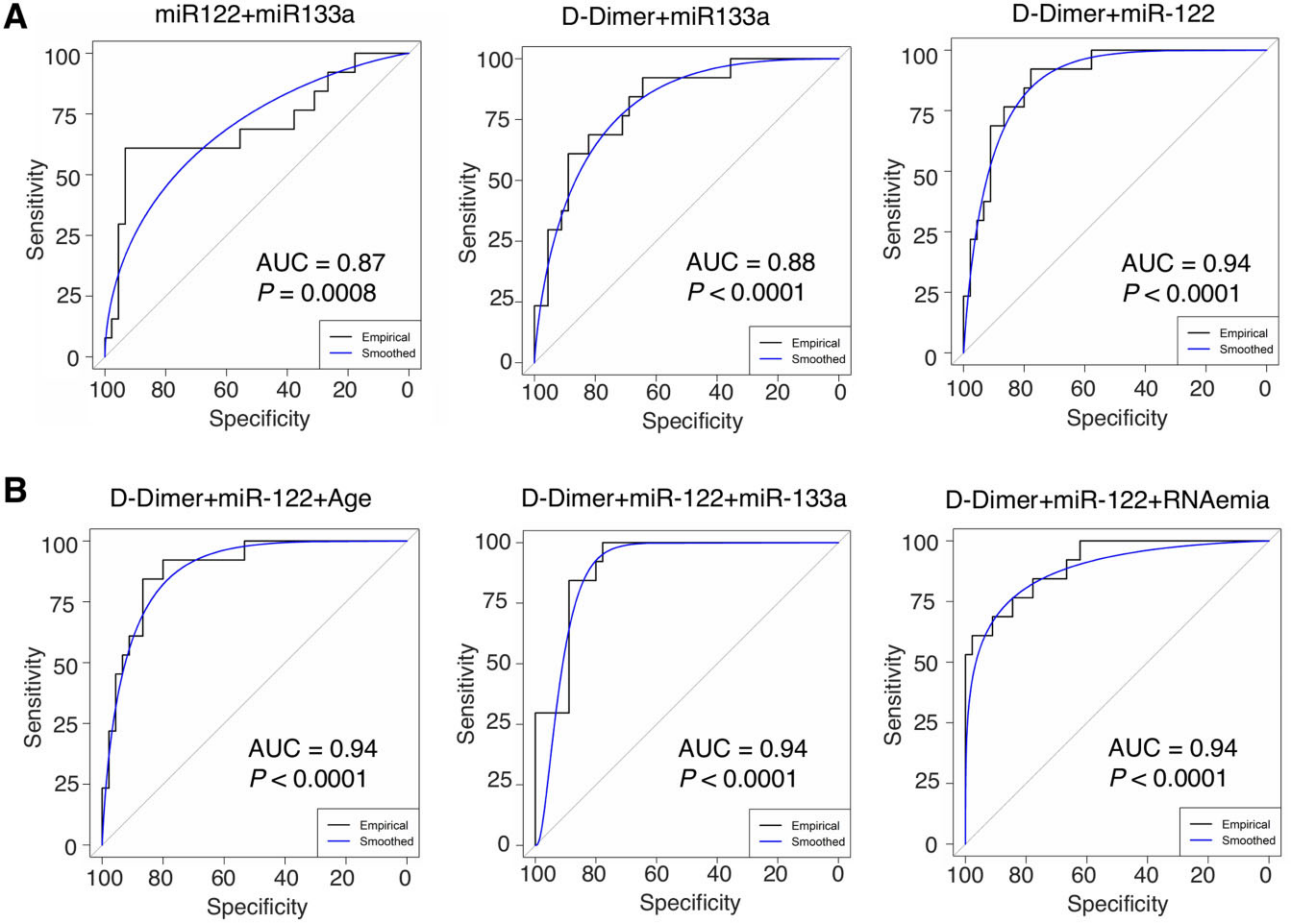
COVID-19 severity classification. (*A* and *B*) ROC plots for the best three binary (*A*) and best three triplet (*B*) severity signatures are shown. The non-severe cohort (*n *=* *45) consisted of *n *=* *6 mild and *n *=* *39 moderate patients, whilst the severe cohort consisted of *n *=* *16 patients. All statistical analyses are two-tailed.

Finally, we explored the classification performance of miRNAs for COVID-19 severity and mortality, using binary and triplet combinations of miR-133a and miR-122 with established markers of COVID-19 severity and mortality, i.e. age, SARS-CoV-2 RNAemia, BMI, D-dimer, and troponin T.^12^^,^^36^^,^^37^ None of the miRNAs measured by RT–qPCR associated with SARS-CoV-2 RNAemia (*Figures 4B and 5B* and Supplementary material online, *Figure S3*). As single variables, RT–qPCR measurements of miR-122 (AUC = 0.75, *P *=* *0.003) and miR-133a (AUC = 0.79, *P *=* *0.014) outperformed RNAemia, age, BMI, and troponin T in classifying patients into severe (*n *=* *16) and non-severe (i.e. mild or moderate, *n *=* *45) COVID-19 cases, whilst D-dimer had a higher AUC of 0.84 (*P *=* *0.003, *Figure 6* and Supplementary material online, *Table S11*). Binary and triplet combinations provided a further uplift in classification performance, with ‘D-dimer + miR-122’ being the best binary signature (AUC = 0.94, *P *<* *0.0001) and ‘D-dimer + miR-122 + RNAemia’ being the best triplet signature (AUC = 0.94, *P *<* *0.0001).

**Figure 7:**
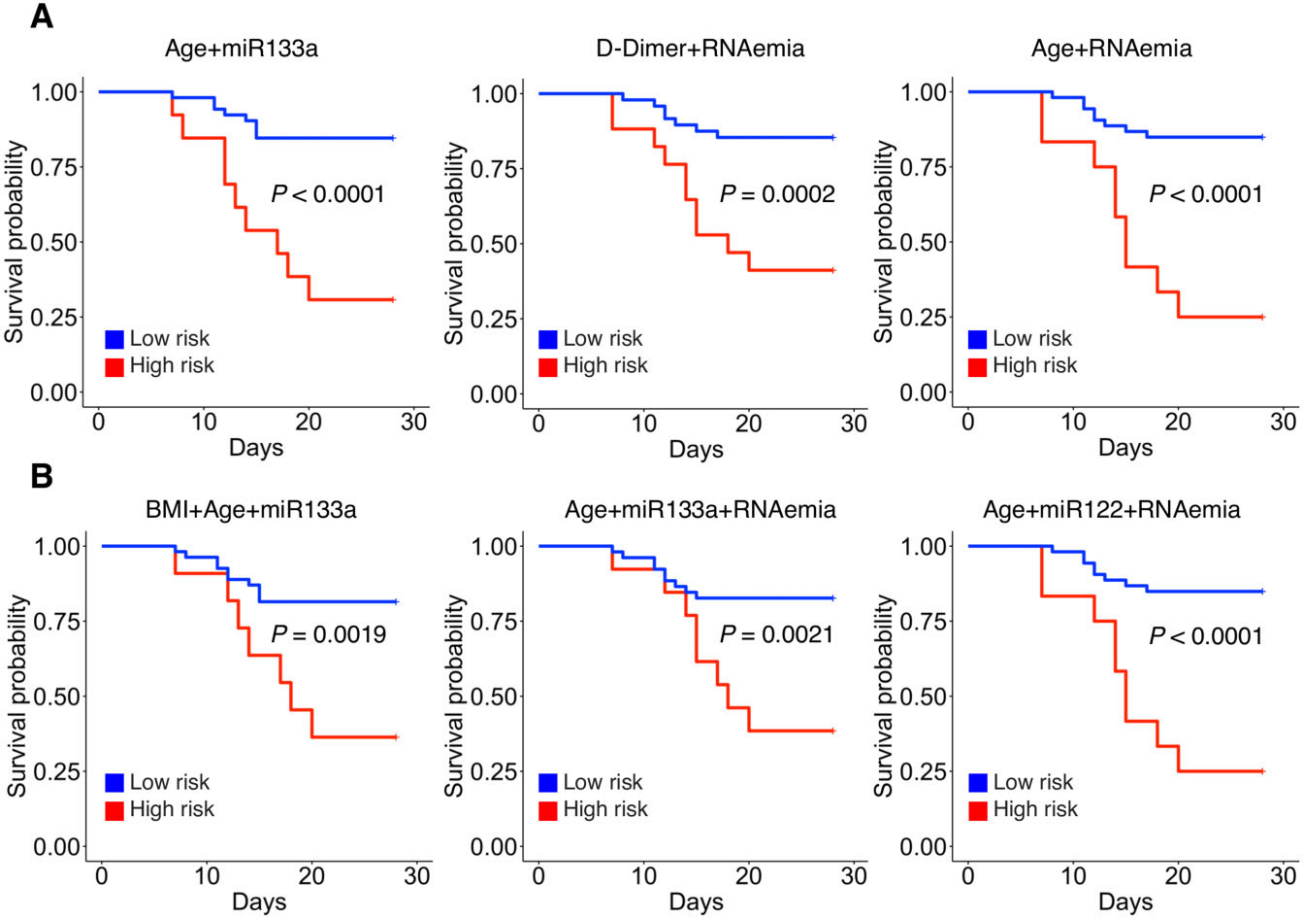
The 28 day ICU mortality in COVID-19 patients. (*A* and *B*) Kaplan–Meier plots for the best three binary (*A*) and best three triplet signatures (*B*) for 28 day ICU mortality classification are shown. Low- and high-risk groups in the Kaplan–Meier analysis are based on the default 0.5 threshold of the logistic regression. The outcome analysis is based on *n *=* *17 COVID-19 ICU non-survivors and *n *=* *48 ICU survivors. All statistical analyses are two-tailed.

In contrast, age, RNAemia, and troponin T were better in predicting 28 day ICU mortality than miRNAs (*Figure 7* and Supplementary material online, *Table S12*). Here, the binary combination of ‘age + RNAemia’ achieved an AUC of 0.85 (*P *<* *0.0001). Only the binary combination ‘miR-133a + age’ achieved a similarly high classification performance (AUC = 0.82, *P *<* *0.0001). When triplet combinations were explored, ‘age + miR-122 + RNAemia’ provided an uplift to ‘age + RNAemia’ and showed the highest overall classification performance (AUC = 0.89, *P *<* *0.0001).

## 4. Discussion

In this study, we identified miRNA-based signatures of disease severity and 28 day ICU mortality in COVID-19 patients. Using RNA-Seq of small RNAs, we identified miRNAs of different cell- and tissue origin that differed in patients with increasing COVID-19 severity. Several of these miRNAs have previously been implicated in cardiovascular^4^^,^^5^^,^^38^ and metabolic diseases^6^^,^^32^ and have also been associated with outcomes and organ dysfunction in critically ill patients (Supplementary material online, *Table S1*). Using RT–qPCR, we associated the best performing miRNAs with 28 day mortality in an ICU cohort of COVID-19 patients.

Among the miRNAs associated with COVID-19 severity were lung-derived miR-187^39^^,^^40^ as well as cardiomyocyte-derived miR-208b,^5^ which were both only detectable by RT–qPCR in patients with severe disease. Endothelial-derived miR-126^4^^,^^32^ levels also rose with severity, in line with the reported systemic activation of the endothelium in COVID-19.^41^ Similarly, all platelet-derived miRNAs, i.e. miR-21, miR-197, and miR-223,^4^ rose with COVID-19 severity. This is consistent with the findings for miR-21 reported by Garg *et al*.^18^^,^^42^^,^^43^ Circulating platelet-derived miRNA levels are known to be associated with platelet reactivity and to be responsive to antiplatelet therapy.^4^^,^^33^^,^^34^ The broad up-regulation of platelet-derived miRNAs therefore likely reflects the reported prothrombotic state in COVID-19.^44^ The most pronounced change of organ-specific miRNAs in severe COVID-19 was seen with myomiR miR-133a^5^^,^^9^^,^^31^ and liver-derived miR-122,^6^ which also associated with 28 day ICU mortality. This is in line with recent proteomics data: Filbin *et al.*^13^ reported that liver protein signatures are reduced with increasing COVID-19 severity, whilst protein signatures related to myocyte damage increased in the circulation.^13^ Similar to miR-133a, elevated baseline levels of myocyte proteins also related to poor survival.^13^ Thus, there is concordance between protein and miRNA biomarkers. Compared to age and SARS-CoV-2 RNAemia,^12^ miR-122 and miR-133a performed well as severity markers but were inferior in outcome prediction. The advantage of miRNAs, however, is their consistent detectability in patients with lower COVID-19 severity and less dependence on sampling time. In contrast, RNAemia is only detectable in the early stages of patients with severe COVID-19.^12^ Another important feature of miR-122^6^ and miR-133a^5^^,^^9^^,^^31^^,^^45–48^ is their exquisite tissue specificity that offers insight into the underlying pathophysiology. Moreover, the integration of miRNAs into biomarker signatures may improve the performance of established biomarkers, as demonstrated for binary and triplet combinations with D-dimer, Troponin T, SARS-CoV-2 RNAemia, age, and BMI.^12^^,^^36^^,^^37^

MiR-133a has been previously shown to be elevated in sepsis patients^45^^,^^47^ and to predict mortality in critically ill patients (*n *=* *223).^45^ A rise of miR-133a is also observed in murine sepsis models.^45–47^ Similarly, myocardial injury^5^^,^^9^^,^^31^ and chronic obstructive pulmonary disease (COPD)^48^ are known to increase circulating miR-133a levels. Accordingly, among critically ill patients without sepsis, miR-133a levels were highest in patients with cardiopulmonary diseases.^45^ Interestingly, we found miR-133a levels to be inversely correlated to neutrophil counts. MPO, a neutrophil activation marker associated with cardiovascular outcomes,^49^ endothelial cell apoptosis,^50^ and matrix metalloprotease activation,^51^ was positively correlated to miR-133a. Thus, neutrophil degranulation and extravasation resulting in myocyte damage is a likely cause for the rise of circulating miR-133a.^52–54^ Moreover, there is evidence that neutrophils may be a secondary source of miR-133a in the circulation.^55^

In our recent proteomics analysis of inflammation signatures in endotoxaemia, time course analysis showed a time-dependent increase in MPO upon LPS treatment.^35^ Neutrophil-derived proteins were also deposited in the vessel wall upon LPS injection.^35^ A negative correlation with miR-133a levels was observed for pulmonary SFTPB, a lung protein responsible for alveolar stability. Mendelian randomization studies have shown that single-nucleotide polymorphisms associated with higher serum surfactant protein levels impart a lower risk for COPD and lung function decline.^56^^,^^57^ In COPD patients, miR-133a levels are known to be increased.^48^ Given that SFTPB is not a predicted target of miR-133a,^58–60^ the inverse association between SFTPB and miR-133a is likely a consequence of the same pathological mechanism that leads to their increase in blood rather than a functional relationship.

In contrast to miR-133a, levels of miR-122 were reduced in non-survivors. This is consistent with the notion that early depression of liver function is associated with poor outcome in COVID-19 patients.^11–15^ Moreover, miR-122 levels were positively associated with liver-derived negative APPs, which decrease upon infection to favour synthesis of positive APPs. MiR-122 was inversely associated with the latter. In our recent plasma proteomics study to assess inflammation signatures in endotoxemia,^35^ positive APPs increased at 24 h following neutrophil degranulation within 6 h post-endotoxin administration.^35^ Recent evidence suggests that miR-122 might be involved in both the hepatic and pulmonary host response to viral infections. For instance, a recent study surprisingly detected miR-122 in the lungs and reported that intrapulmonary miR-122 increases neutrophilic inflammation during rhinovirus infection.^61^ In hepatocytes, miR-122 also plays an important role for innate immunity during viral infections.^62^ MiR-122 promotes type I and II interferon (IFN) expression in response to a variety of viral nucleic acids.^62^ Patients with severe COVID-19 present with a paradoxical antiviral immune response: their IFN-response is delayed or suppressed and often preceded by an excessive pro-inflammatory response that aggravates disease.^63^^,^^64^ Suppression of liver metabolism in critically ill COVID-19 patients as indicated by lower liver-derived miRNA levels in non-survivors,^16^ and elderly patients in particular, might be a distinguishing pathophysiological feature of COVID-19 compared with other critical conditions, such as sepsis or acute respiratory distress syndrome.

Studies of miR-122 in sepsis^65^ or acute respiratory distress syndrome^66^ reported an increase in non-survivors, which has been attributed to acute liver injury.^67^ Similarly, miR-122 is part of a recently reported prognostic model for acetaminophen-induced acute liver failure.^68^ We believe that the discrepancy of miR-122 directionality can be attributed to the fact that miR-122 levels are a readout of two distinct pathophysiological processes, i.e. liver injury vs. liver metabolism. Our previous findings in the community-based Bruneck study revealed elevated miR-122 to be a strong predictor for metabolic syndrome over a 10–15 year observation period.^69^ Similar to COVID-19 patients,^6^ miR-122 levels were highly correlated to many liver-derived plasma or serum proteins.^69^

In conclusion, baseline levels of myocyte-derived miR-133a and liver-derived miR-122 are associated with COVID-19 severity and 28 day ICU mortality, reflecting inflammation-induced myocyte damage and the hepatic acute phase response. Based on the comparison to protein correlations in COVID-19, miR-122 shows a trajectory that is similar to negative APPs. Future studies are needed to clarify whether miR-133a or miR-122 measurement have the potential to aid in prognosis assessment by monitoring organ damage and resolution of inflammation that might inform treatment decisions.

## Supplementary material

Supplementary material is available at *Cardiovascular Research* online.

## Authors’ contributions

C.G., K.K., K.T., S.A.B., L.E.S., J.D.E., A.M.S., A.C.H., M.H., M.S.-H., and M.M. contributed to the study design, data interpretation, and writing of the manuscript. C.G., K.K., K.T., A.B.D., S.A.B., L.E.S., C.C., and M.H. contributed to the laboratory data generation and analysis. A.N., M.F., M.J.W.M., K.O.G., G.A., S.N., S.F.M., F.T., B.S., B.M., L.T., S.E., and M.S.-H. contributed to participant recruitment, sample collection, sample processing, and clinical data collection. All authors reviewed the manuscript.

## Supplementary Material

cvab338_Supplementary_Data

## Notes

Translational perspective Adding biomarkers to conventional scores for illness severity and mortality could improve prognostic performance in COVID-19 patients. Circulating miRNAs are emerging as promising biomarkers with tissue-specific origins but have only sparsely been investigated in COVID-19. We quantified circulating miRNAs of different tissue origin in COVID-19 patients, identifying several miRNAs of the cardiometabolic system to be associated with severity. Myocyte-derived miR-133a and liver-derived miR-122 also associated with mortality. Through longitudinal proteomics measurements, we related myomiR miR-133a release to neutrophil activation and miR-122 release to the hepatic acute phase response. Our findings highlight key pathophysiological changes and provide first evidence on the performance of miRNA biomarkers in COVID-19.
