# Supplementary material for: Association of cardiometabolic microRNAs with COVID-19 severity and mortality
Source: Cardiovasc Res. 2021 Nov 10;118(2):461–74. doi: 10.1093/cvr/cvab338 (PMC8689968; doi:10.1093/cvr/cvab338)
Supplement: cvab338_Supplementary_Data [file cvab338_supplementary_data.docx]

**Supplementary material**

**Association of cardiometabolic microRNAs with COVID-19 severity and mortality**

Short title: Circulating cardiometabolic microRNAs in COVID-19

Clemens Gutmann^1^, Kseniya Khamina^2^, Konstantinos Theofilatos^1^, Andreas B. Diendorfer^2^, Sean A. Burnap^1^, Adam Nabeebaccus^1,3^, Matthew Fish^4,5^, Mark J.W. McPhail^3,6,7^, Kevin O'Gallagher^1,3^, Lukas E. Schmidt^1^, Christian Cassel^1^, Georg Auzinger^3^, Salvatore Napoli^6^, Salma F. Mujib^7^, Francesca Trovato^3,6,7^, Barnaby Sanderson^5^, Blair Merrick^8^, Roman Roy^3^, Jonathan D. Edgeworth^4,8^, Ajay M. Shah^1,3^, Adrian C. Hayday^4,9^, Ludwig Traby^10^, Matthias Hackl^2^, Sabine Eichinger^11^, Manu Shankar-Hari^4,5,*^, Manuel Mayr^1,*^

^1^King's College London British Heart Foundation Centre, School of Cardiovascular Medicine and Sciences, London, UK.

^2^TAmiRNA GmbH, Vienna, Austria.

^3^King's College Hospital NHS Foundation Trust, London, UK.

^4^Peter Gorer Department of Immunobiology, School of Immunology and Microbial Sciences, King’s College London, London, UK.

^5^Department of Intensive Care Medicine, Guy's and St Thomas' NHS Foundation Trust, London, UK.

^6^Department of Inflammation Biology, School of Immunology and Microbial Sciences, Faculty of Life Sciences and Medicine, King's College London, London, UK.

^7^Institute of Liver Studies, King’s College Hospital, London, UK.

^8^Centre for Clinical Infection and Diagnostics Research, Department of Infectious Diseases, Gu’s and St Thomas’ NHS Foundation Trust & King’s College London, London, UK.

^9^The Francis Crick Institute, London, UK.

^10^Department of Medicine I, Division of Infectious Diseases and Tropical Medicine, Medical University of Vienna, Vienna, Austria.

^11^Department of Medicine I, Division of Haematology and Hemostaseology

Medical University of Vienna, Vienna, Austria.

*Corresponding authors: Prof. Manu Shankar-Hari, Department of Intensive Care Medicine, Guy’s and St Thomas’ NHS Foundation Trust, London, UK. [manu.shankar-hari@kcl.ac.uk](mailto:manu.shankar-hari@kcl.ac.uk) and Prof. Manuel Mayr, King's College London British Heart Foundation Centre, School of Cardiovascular Medicine and Sciences, London, UK. [manuel.mayr@kcl.ac.uk](mailto:manuel.mayr@kcl.ac.uk)

**
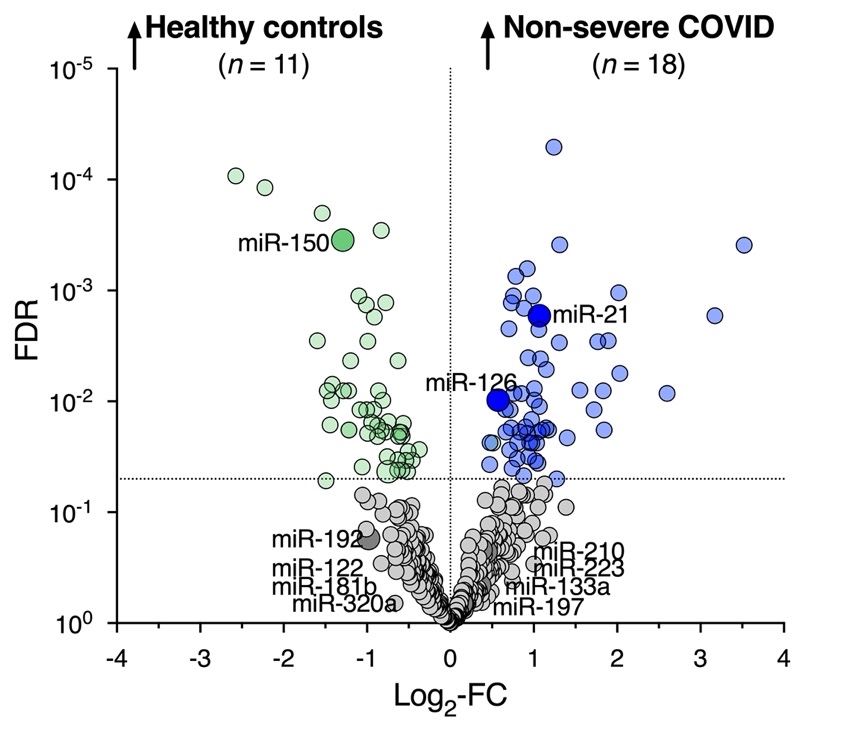
**

**Figure S1.** Volcano plot showing differential expression of circulating miRNAs between healthy controls (*n* = 11) and non-severe COVID-19 patients (*n* = 18). Highlighted are miRNAs that have previously been attributed a role in critically ill patients or are miRNAs with a specific organ origin (*Table S1*). Differential expression analysis of RNA-Seq data was performed using edgeR and applying the independent filtering method of DESeq2 to remove low abundant miRNA to optimize the Benjamini-Hochberg false discovery rate (FDR) correction.

**
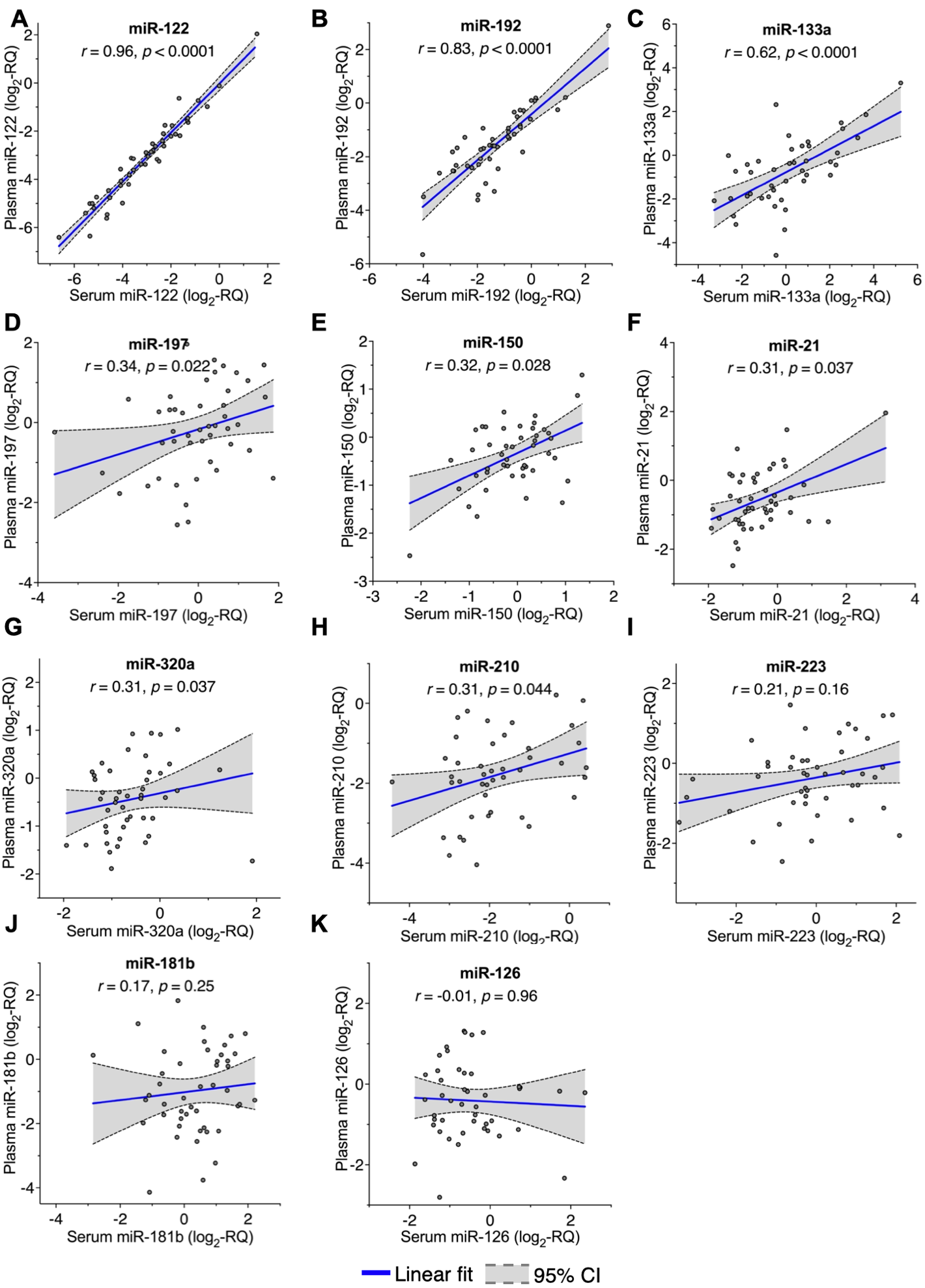
**

**Figure S2.** Spearman correlation between serum and plasma measurements of miRNAs in samples of the King’s College Hospital ICU cohort where both plasma and serum were available (*n* = 13 patients, *n* = 49 samples). Displayed are miRNA correlations from high to low correlation strength: miR-122 (*A*), miR-192 (*B*), miR-133a (*C*), miR-150 (*D*), miR-21 (*E*), miR-210 (*F*), miR-197 (*G*), miR-223 (*H*), miR-320a (*I*), miR-181b (*J*) and miR-126 (*K*). Detectability of miR-187 (2.2%), miR-208b (4.4%) and miR-124 (30.4%) was low in serum and in poor agreement (Cohen’s kappa) with plasma measurements (miR-187 *κ* = 0.09, 95%CI [-0.16-0.33]; miR-208 *κ* = -0.04, 95%CI [-0.12-0.03]; miR-124 *κ* = 0.09, 95%CI [-0.16-0.34]).

**Figure S3.** SARS-CoV-2 RNAemia frequency and association with miRNA levels in plasma. (A) RNAemia frequency in COVID-19 patients with mild (n = 6), moderate (n = 39) disease and severe disease (n = 16). Statistical significance in (A) was determined using the Chi-square test. (B-C) Baseline plasma miRNA expression levels in COVID-19 ICU patients who tested positive or negative for SARS-CoV-2 RNAemia at baseline (B) or in at least one of two tests within six days of ICU admission (C). Significance in (B-C) was determined using multiple unpaired, two-tailed student t-tests and applying the Benjamini and Hochberg’s correction.

**
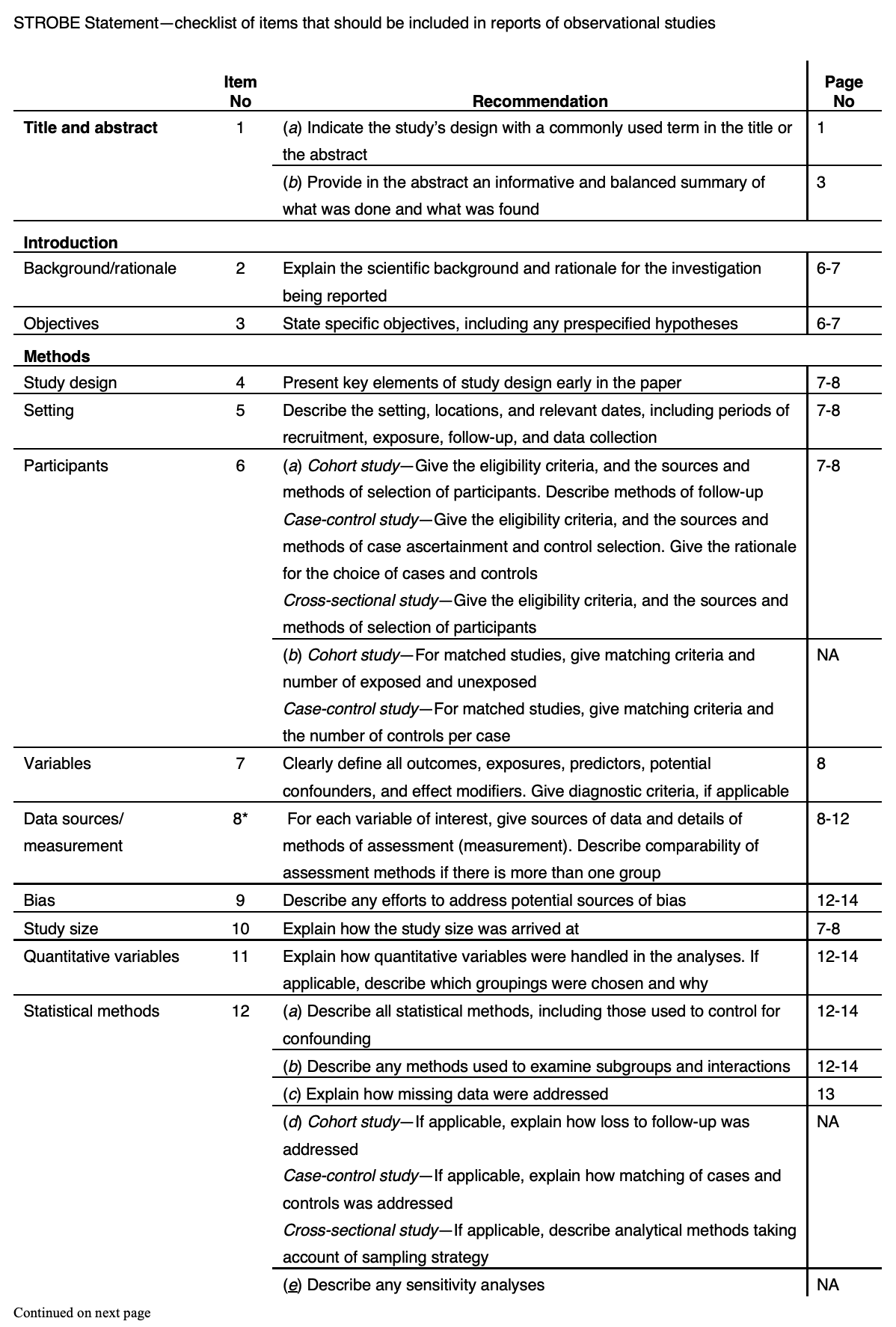
**

**Figure S4.** STROBE checklist (1/2).

**
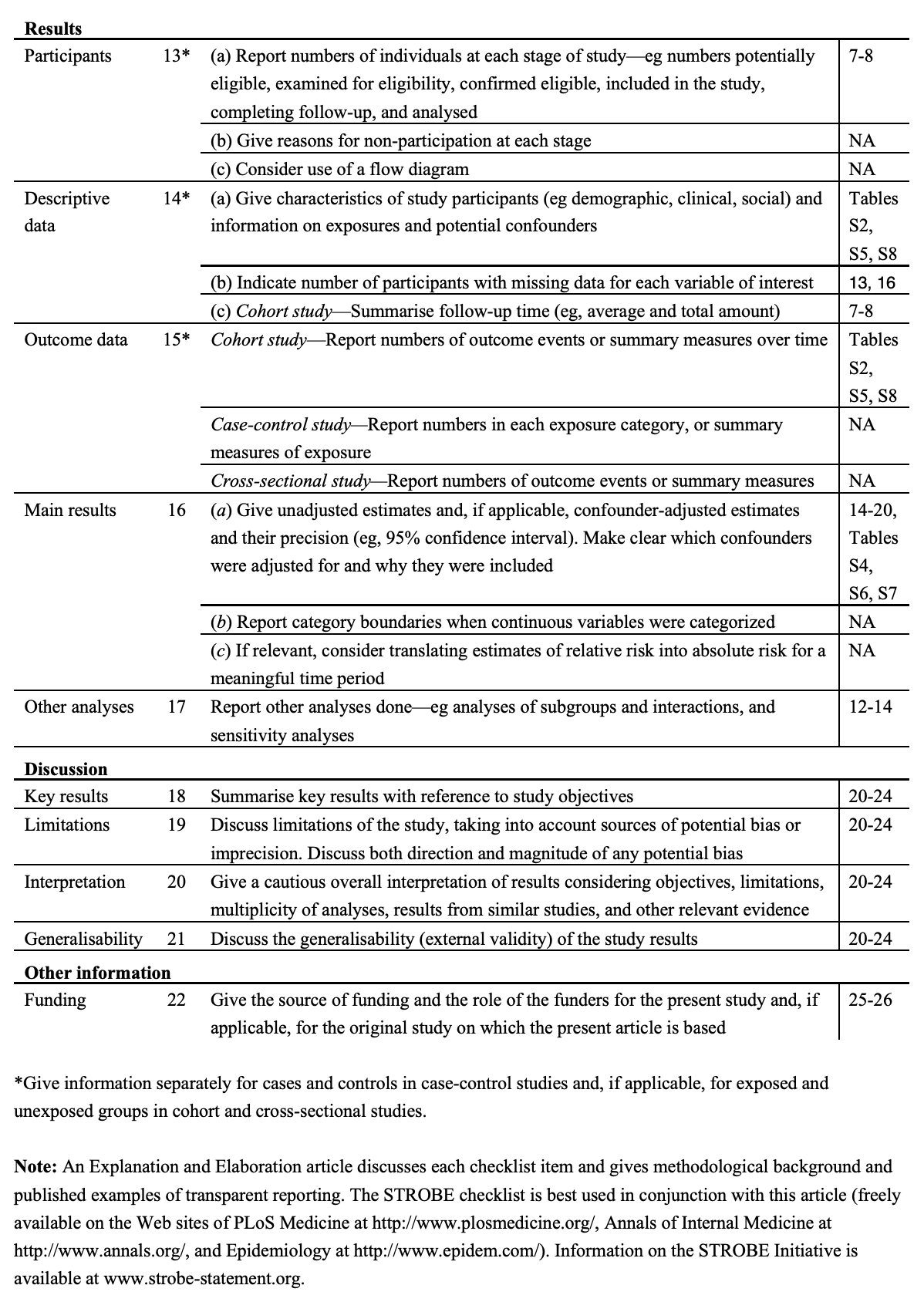
**

**Figure S5.** STROBE checklist (2/2).

**Table S1. Literature-based selection of miRNAs from different tissue-origin and disease association.**

| **Target** | **Predominant cell / tissue origin** | **Pathophysiological association** |
| --- | --- | --- |
| miR-210-3p | Liver, Kidney^1^ | Marker of acute kidney failure^1^ |
| miR-122-5p | Liver^2^ | Marker of acute liver failure^2^ |
| miR-192-5p | Liver^2^ | Marker of non-alcoholic fatty liver disease^2^ |
| miR-320a-3p | Kidney, ubiquitous^1^ | Marker of acute kidney failure^1^ |
| miR-126-3p | Endothelium, platelets^3,4^ | Activation of endothelium and platelets, cardiovascular disease^3,4^ |
| miR-21-5p | Platelets, ubiquitous^3,5,6^ | Activation of platelets, cardiovascular disease^3,5,6^ |
| miR-223-3p | Platelets^3,5,6^ | Activation of platelets, cardiovascular disease^3,5,6^ |
| miR-197-3p | Platelets^3,5,6^ | Activation of platelets, cardiovascular disease^3,5,6^ |
| miR-150-5p | Leukocytes, platelets^7,8^ | Marker of sepsis^7,8^ |
| miR-133a-3p | Myocytes^9–16^ | Marker^9,10^ and functional role^11^ in sepsis, marker of COPD,^12^ marker of myocardial damage^13–16^ |
| miR-181b-5p | Lung^17,18^ | Marker of acute lung injury^17,18^ |
| miR-187-5p | Lung^17,18^ | Marker of acute lung injury^17,18^ |
| miR-208b-3p | Cardiomyocytes^13^ | Marker of myocardial damage^13^ |
| miR-124-3p | Neurons^19^ | Marker of neurological outcome^19^ |

**Table S2. Clinical characteristics of COVID-19 patients and healthy volunteers for whom baseline plasma samples were used for RNA-Seq.**

| **Clinical Characteristics** | **Healthy controls (*n*=11)** | **Non-Severe**  **(*n*=18)** | **Severe**  **(*n*=18)** | ***P* value** |
| --- | --- | --- | --- | --- |
| Non-Survivors 28 days after hospitalization (%) | 0 | 0 | 16.7 | **0.02** |
| **Demographics** |  |  |  |  |
| Age (years) | 40.0 (30.0, 46.0) | 55.0 (36.0, 66.0) | 58.0 (39.0, 66.0) | 0.10 |
| BMI (kg/m^2^) | - | 26.8 (24.3, 30.8) | 30.1 (27.7, 36.7) | 0.07 |
| Sex (% male) | 42.0 | 56.0 | 42.0 | 0.37 |
| **Pre-existing conditions** |  |  |  |  |
| Active smoker (%) | - | 11.1 | 11.1 | 0.99 |
| Adiposity (%) | - | 33.3 | 50.0 | 0.49 |
| Atrial fibrillation (%) | - | 0 | 5.6 | 0.31 |
| Autoimmune dis. (%) | - | 0 | 0 |  |
| Cancer – active (%) | - | 0 | 5.6 | 0.31 |
| Cancer – non-active (%) | - | 5.6 | 5.6 | 0.99 |
| Cardiovasc. dis. (%) | - | 22.2 | 11.1 | 0.37 |
| Chronic infect. dis. (%) | - | 0 | 0 |  |
| COPD (%) | - | 5.6 | 5.6 | 0.99 |
| Diabetes (%) | - | 22.2 | 22.2 | 0.99 |
| Endocrine disorder (%) | - | 11.1 | 0 | 0.15 |
| Hypercholesteremia (%) | - | 11.1 | 5.6 | 0.55 |
| Hypertension (%) | - | 38.8 | 33.3 | 0.73 |
| Kidney disease (%) | - | 16.7 | 0 | 0.07 |
| Liver disease (%) | - | 0 | 11.1 | 0.15 |
| Neurological dis. (%) | - | 5.6 | 27.8 | 0.07 |
| Other lung disease (%) | - | 11.1 | 16.7 | 0.63 |
| Pregnancy (%) | - | 0 | 5.6 | 0.31 |
| Psychiatric dis. (%) | - | 0 | 11.1 | 0.15 |
| VTE (%) | - | 0 | 16.7 | 0.07 |
| **Pre-medication** |  |  |  |  |
| None (%) | - | 33.3 | 27.8 | 0.72 |
| Anticoagulant (%) | - | 0.0 | 16.7 | 0.07 |
| Antidepressant (%) | - | 38.9 | 22.2 | 0.28 |
| Antidiabetic drug (%) | - | 27.8 | 22.2 | 0.70 |
| Antihypertensive (%) | - | 38.9 | 38.9 | 0.99 |
| Antiplatelet therapy (%) | - | 22.2 | 11.1 | 0.37 |
| ACE inhibitor (%) | - | 5.6 | 22.2 | 0.15 |
| AT1 inhibitor (%) | - | 16.7 | 11.1 | 0.63 |
| Beta-blocker (%) | - | 16.7 | 16.7 | 0.99 |
| Calcium channel bl. (%) | - | 16.7 | 11.1 | 0.63 |
| Diuretic (%) | - | 22.2 | 11.1 | 0.37 |
| PPI (%) | - | 33.3 | 33.3 | 0.99 |
| Statins (%) | - | 27.8 | 11.1 | 0.21 |
| Steroids (%) | - | 22.2 | 11.1 | 0.37 |
| Thyroid hormone (%) | - | 16.7 | 5.6 | 0.29 |
| Vasodilator (%) | - | 0.0 | 5.6 | 0.31 |
| **Therapy during inpatient care** |  |  |  |  |
| Antivirals (%) | - | 16.7 | 72.2 | **0.0008** |
| Convalesc. plasma(%) | - | 0 | 5.6 | 0.31 |
| Corticosteroids (%) | - | 5.6 | 83.3 | **<0.0001** |
| I.v. immunogl. (%) | - | 0 | 16.7 | 0.07 |
| **Acute Care Parameters** |  |  |  |  |
| FiO_2_ (fraction of 1) | - | - | 0.6 (0.5, 0.7) |  |
| Heart rate (bpm) | - | - | 85.0 (79.0, 96.0) |  |
| PaO_2_ (fraction of 1) | - | - | 77.1 (68.0, 103.0) |  |
| PaO_2_/FiO_2_ ratio | - | - | 147.1 (95.4, 196.1) |  |
| pH | - | - | 7.4 (7.3, 7.5) |  |
| Resp. rate | - | - | 20.0 (16.0, 28.0) |  |
| Temperature (°C) | - | - | 37.2 (36.1, 37.7) |  |
| **Type of Oxygen support** |  |  |  |  |
| None (%) | 100 | 16.7 | 0 | **<0.0001** |
| Low flow insuffl. (%) | 0 | 16.6 | 5.6 | 0.25 |
| High flow insuffl. (%) | 0 | 0 | 16.6 | 0.08 |
| Non-invasive ventil. (%) | 0 | 0 | 16.6 | 0.08 |
| Invasive ventil. (%) | 0 | 0 | 61.1 | **<0.0001** |
| ECMO (%) | 0 | 0 | 0 |  |
| **Blood Biochemistry** |  |  |  |  |
| Albumin (g/L) | - | 37.8 (33.3, 44.7) | 29.6 (27.7, 30.9) | **<0.0001** |
| ALT (IU/L) | 29.0 (17.0, 40.0) | 21.0 (17.3, 43.5) | 52.0 (33.2, 65.5) | 0.06 |
| aPTT (seconds) | 34.9 (32.7, 37.6) | 39.7 (36.2, 42.4) | 37.7 (32.1, 42.9) | 0.26 |
| AT (%) | - | 103.0 (92.0, 106.0) | 91.5 (75.8, 108.0) | 0.20 |
| Bilirubin (µmol/L) | - | 7.8 (5.4, 9.9) | 10.1 (8.5, 18.7) | 0.06 |
| BUN (mg/dL) | - | 15.1 (9.6, 22.9) | 25.8 (17.8, 30.2) | **0.006** |
| CK (U/L) | - | 63.0 (40.3, 92.5) | 169.5 (72.3, 350.8) | **0.03** |
| CRP (mg/L) | 0.9 (0.5, 2.9) | 41.2 (16.2, 70.3) | 141.4 (71.9, 219.7) | **<0.0001** |
| Creatinine (µmol/L) | 75.6 (70.1, 83.9) | 83.5 (75.4, 98.8) | 82.2 (59.9, 95.0) | 0.73 |
| D-dimer (µg/ml) | - | 0.49 (0.35, 0.96) | 2.08 (0.63, 4.93) | **0.02** |
| Ferritin (µg/L) | - | 298 (148, 579) | 1483 (670, 2110) | **0.0006** |
| Fibrinogen (mg/dL) | 246.0 (205.5, 312.8) | 487.0 (400.8, 584.0) | 568.0 (478.0, 636.8) | **0.0007** |
| Hematocrit (%) | - | - | 36.6 (29.3, 41.1) |  |
| Hemoglobin (g/dL) | 14.3 (13.9, 14.6) | 13.2 (11.9, 15.0) | 11.1 (9.1, 12.7) | **0.0003** |
| Interleukin 6 (pg/ml) | - | 14.9 (8.2, 45.3) | 60.9 (21.6, 156.0) | **0.004** |
| LDH (U/L) | 176.0 (172.0, 180.0) | 266.0 (199.0, 343.0) | 336.0 (256.3, 451.3) | **0.02** |
| NT-pro-BNP (pg/mL) | - | 103.9 (31.1, 456.3) | 280.0 (73.3, 872.0) | 0.36 |
| Procalcitonin (ng/ml) | - | 0.12 (0.04, 0.15) | 0.34 (0.09, 0.62) | 0.05 |
| Troponin T (ng/L) | - | 9.0 (5.0, 25.5) | 9.5 (4.0, 35.3) | 0.99 |
| TT (seconds) | - | 18.5 (16.8, 19.8) | 19.2 (18.3, 20.4) | 0.15 |
| **Blood Count** |  |  |  |  |
| Lymphocytes (10^9^/l) | 1.8 (1.6, 2.1) | 1.1 (0.7, 1.3) | 0.5 (0.4, 0.7) | **<0.0001** |
| Monocytes (10^9^/l) | - | 0.4 (0.3, 0.7) | 0.5 (0.3, 0.6) | 0.50 |
| Neutrophils (10^9^/l) | 3.6 (2.8, 4.5) | 2.9 (2.0, 5.4) | 7.3 (4.9, 9.2) | **0.0002** |
| Neutroph./Lymph. ratio | 1.7 (1.5, 2.3) | 3.3 (1.7, 5.3) | 20.5 (5.9, 27.5) | **<0.0001** |
| Platelets (10^9^/l) | 214.0 (188.0, 244.0) | 208 (157, 257) | 274 (158, 339) | 0.41 |
| White cell count(10^9^/l) | 6.6 (5.2, 7.2) | 4.8 (3.8, 6.4) | 8.5 (7.6, 10.9) | **0.0005** |

Abbreviations: ACE inhibitor: angiotensin converting enzyme inhibitor, ALT: alanine aminotransferase, aPTT: activated partial thromboplastin time, AT: antithrombin, BMI: body mass index, BUN: blood urea nitrogen, CK: creatine kinase, COPD: chronic obstructive pulmonary disease, CRP: C-reactive protein, ECMO: extracorporeal membrane oxygenation, FiO_2_: fraction of inspired oxygen, i.v.: intravenous, LDH: lactate dehydrogenase, PPI: proton pump inhibitor, TT: thrombin time, VTE: venous thromboembolism. “–“ indicates that this variable was not collected. For continuous variables, medians and interquartile ranges are shown, and Mann-Whitney U tests were performed to determine significance between two groups, whilst Kruskal-Wallis tests were used when three groups were compared. For binary variables, percentages are shown, and Chi-square tests were performed to determine statistical significance.

**Table S3. List of miRNAs assessed by RNA-Seq in plasma of healthy controls (*n* = 11) and COVID-19 patients with severe disease (*n* = 18), ranked by logarithmic fold change (Log_2_-FC).**

| **miRNA** | **Log_2_-FC** | ***P* value** | **FDR** |
| --- | --- | --- | --- |
| miR-4454 | 4.27 | **0.0001** | **0.0003** |
| miR-10396b-5p | 4.24 | **0.0001** | **0.0003** |
| miR-10396a-5p | 4.24 | **0.0001** | **0.0003** |
| miR-1290 | 3.69 | **0.0002** | **0.0010** |
| miR-3960 | 2.92 | **0.0002** | **0.0009** |
| miR-193b-3p | 2.73 | **0.0005** | **0.0018** |
| miR-4488 | 2.69 | **0.0001** | **0.0006** |
| miR-133a-3p | 2.35 | **0.0045** | **0.0115** |
| miR-122-3p | 2.27 | **0.0039** | **0.0101** |
| miR-34a-5p | 2.22 | **0.0005** | **0.0018** |
| miR-885-5p | 2.08 | **0.0018** | **0.0055** |
| miR-1246 | 2.04 | **0.0199** | **0.0401** |
| miR-199b-5p | 2.04 | **<0.0001** | **<0.0001** |
| miR-210-3p | 1.73 | **0.0002** | **0.0007** |
| miR-122-5p | 1.67 | 0.0732 | 0.1212 |
| miR-148a-3p | 1.60 | **0.0031** | **0.0085** |
| miR-574-3p | 1.50 | **0.0002** | **0.0009** |
| miR-21-3p | 1.44 | **<0.0001** | **<0.0001** |
| miR-27b-3p | 1.41 | **0.0075** | **0.0170** |
| miR-21-5p | 1.34 | **<0.0001** | **<0.0001** |
| miR-223-5p | 1.33 | **<0.0001** | **<0.0001** |
| miR-33a-5p | 1.26 | **<0.0001** | **<0.0001** |
| miR-22-3p | 1.26 | **0.0026** | **0.0073** |
| miR-15a-5p | 1.26 | **<0.0001** | **0.0001** |
| miR-130a-3p | 1.24 | **<0.0001** | **<0.0001** |
| miR-29c-3p | 1.20 | **<0.0001** | **0.0003** |
| miR-32-5p | 1.15 | **0.0006** | **0.0020** |
| miR-141-3p | 1.13 | **0.0298** | 0.0574 |
| miR-130b-3p | 1.12 | **<0.0001** | **<0.0001** |
| miR-505-3p | 1.10 | **0.0030** | **0.0083** |
| miR-532-3p | 1.04 | **0.0004** | **0.0015** |
| miR-199a-5p | 1.03 | **0.0027** | **0.0075** |
| miR-497-5p | 1.02 | **0.0019** | **0.0057** |
| miR-17-5p | 1.01 | **<0.0001** | **0.0002** |
| miR-324-5p | 1.00 | **<0.0001** | **<0.0001** |
| miR-30a-5p | 0.97 | **0.0080** | **0.0180** |
| miR-22-5p | 0.97 | **0.0073** | **0.0168** |
| miR-99a-5p | 0.96 | 0.1048 | 0.1647 |
| miR-126-5p | 0.93 | **<0.0001** | **0.0001** |
| miR-29a-3p | 0.92 | **0.0054** | **0.0131** |
| miR-324-3p | 0.91 | **<0.0001** | **0.0002** |
| miR-203a-3p | 0.90 | **0.0834** | 0.1364 |
| miR-20b-5p | 0.86 | **0.0021** | **0.0061** |
| miR-7-1-3p | 0.83 | **0.0001** | **0.0007** |
| miR-200a-3p | 0.82 | 0.0664 | 0.1118 |
| miR-223-3p | 0.80 | **0.0056** | **0.0133** |
| miR-142-3p | 0.78 | **0.0003** | **0.0014** |
| miR-345-5p | 0.77 | **0.0155** | **0.0322** |
| miR-192-5p | 0.76 | 0.1817 | 0.2517 |
| miR-200c-3p | 0.75 | 0.0436 | 0.0777 |
| miR-338-3p | 0.75 | **0.0010** | **0.0035** |
| miR-197-3p | 0.74 | **0.0067** | **0.0156** |
| miR-301a-3p | 0.73 | **0.0017** | **0.0053** |
| miR-145-5p | 0.72 | **0.0118** | **0.0256** |
| miR-20a-5p | 0.72 | **0.0039** | **0.0101** |
| miR-193a-5p | 0.72 | 0.1273 | 0.1860 |
| miR-376c-3p | 0.71 | 0.1096 | 0.1680 |
| miR-654-3p | 0.67 | 0.1138 | 0.1730 |
| miR-151b | 0.66 | **0.0088** | **0.0194** |
| miR-378a-3p | 0.65 | 0.0967 | 0.1545 |
| miR-139-5p | 0.65 | 0.0839 | 0.1364 |
| miR-29b-3p | 0.62 | **0.0316** | 0.0597 |
| miR-16-5p | 0.62 | **0.0051** | **0.0126** |
| miR-425-3p | 0.61 | **0.0001** | **0.0004** |
| miR-425-5p | 0.60 | **0.0005** | **0.0018** |
| let-7a-3p | 0.58 | **0.0127** | **0.0274** |
| miR-23b-3p | 0.54 | 0.0534 | 0.0924 |
| miR-152-3p | 0.54 | 0.1222 | 0.1843 |
| miR-424-3p | 0.52 | **0.0345** | 0.0632 |
| miR-28-5p | 0.52 | **0.0327** | 0.0611 |
| miR-874-3p | 0.52 | 0.1240 | 0.1853 |
| miR-941 | 0.50 | **0.0330** | 0.0611 |
| miR-376a-3p | 0.45 | 0.3276 | 0.4148 |
| miR-335-5p | 0.40 | 0.2262 | 0.3000 |
| miR-30c-5p | 0.39 | 0.2254 | 0.3000 |
| miR-27a-3p | 0.39 | **0.0167** | **0.0343** |
| miR-125b-5p | 0.38 | 0.3813 | 0.4691 |
| miR-660-5p | 0.36 | 0.0604 | 0.1037 |
| miR-148b-3p | 0.30 | 0.1601 | 0.2301 |
| miR-17-3p | 0.29 | 0.1644 | 0.2329 |
| miR-361-3p | 0.29 | 0.0939 | 0.1513 |
| miR-24-3p | 0.28 | 0.3089 | 0.4012 |
| miR-221-3p | 0.27 | 0.1739 | 0.2427 |
| miR-23a-3p | 0.27 | 0.1030 | 0.1632 |
| miR-30b-5p | 0.26 | 0.4560 | 0.5501 |
| miR-320c | 0.26 | 0.4785 | 0.5700 |
| miR-106b-5p | 0.24 | 0.4229 | 0.5135 |
| miR-19a-3p | 0.22 | 0.1079 | 0.1680 |
| miR-132-3p | 0.21 | 0.3655 | 0.4587 |
| miR-3615 | 0.20 | 0.3206 | 0.4107 |
| miR-30e-5p | 0.20 | 0.5888 | 0.6673 |
| miR-484 | 0.20 | 0.3283 | 0.4148 |
| miR-19b-3p | 0.20 | 0.2047 | 0.2794 |
| miR-320b | 0.18 | 0.6054 | 0.6779 |
| miR-128-3p | 0.18 | 0.1319 | 0.1912 |
| miR-93-5p | 0.17 | 0.2505 | 0.3299 |
| miR-126-3p | 0.16 | 0.3160 | 0.4075 |
| miR-99b-5p | 0.16 | 0.5182 | 0.6095 |
| miR-151a-5p | 0.15 | 0.5842 | 0.6673 |
| miR-625-3p | 0.13 | 0.6234 | 0.6898 |
| let-7d-3p | 0.12 | 0.6939 | 0.7633 |
| miR-1307-3p | 0.12 | 0.5081 | 0.6014 |
| miR-100-5p | 0.10 | 0.8604 | 0.8966 |
| miR-191-5p | 0.09 | 0.6043 | 0.6779 |
| miR-361-5p | 0.08 | 0.5590 | 0.6533 |
| miR-144-3p | 0.07 | 0.7618 | 0.8331 |
| miR-101-3p | 0.05 | 0.8031 | 0.8581 |
| miR-186-5p | 0.04 | 0.8117 | 0.8605 |
| miR-142-5p | 0.04 | 0.7968 | 0.8563 |
| miR-224-5p | 0.04 | 0.8956 | 0.9103 |
| miR-502-3p | 0.03 | 0.8756 | 0.8997 |
| miR-423-5p | 0.03 | 0.8729 | 0.8997 |
| miR-222-3p | 0.02 | 0.8933 | 0.9103 |
| miR-454-3p | 0.02 | 0.9264 | 0.9364 |
| miR-127-3p | 0.02 | 0.9589 | 0.9589 |
| let-7i-5p | -0.01 | 0.9570 | 0.9589 |
| miR-28-3p | -0.04 | 0.8145 | 0.8605 |
| miR-185-5p | -0.05 | 0.8631 | 0.8966 |
| miR-500a-3p | -0.05 | 0.8252 | 0.8669 |
| miR-151a-3p | -0.09 | 0.5835 | 0.6673 |
| miR-106b-3p | -0.09 | 0.5871 | 0.6673 |
| miR-323b-3p | -0.12 | 0.7864 | 0.8549 |
| miR-196b-5p | -0.12 | 0.6180 | 0.6879 |
| miR-483-5p | -0.14 | 0.7959 | 0.8563 |
| miR-451a | -0.17 | 0.4694 | 0.5627 |
| miR-181b-5p | -0.17 | 0.3799 | 0.4691 |
| miR-15b-5p | -0.21 | 0.5682 | 0.6600 |
| miR-652-3p | -0.24 | 0.1089 | 0.1680 |
| miR-501-3p | -0.26 | 0.3839 | 0.4692 |
| miR-92b-3p | -0.29 | 0.2966 | 0.3879 |
| miR-744-5p | -0.29 | 0.1735 | 0.2427 |
| miR-103a-3p | -0.29 | 0.0642 | 0.1092 |
| miR-30d-5p | -0.34 | 0.2106 | 0.2833 |
| miR-532-5p | -0.35 | **0.0311** | 0.0593 |
| miR-320a-3p | -0.39 | 0.1612 | 0.2301 |
| miR-340-5p | -0.39 | 0.0708 | 0.1182 |
| miR-30a-3p | -0.42 | 0.3725 | 0.4644 |
| miR-140-3p | -0.46 | **0.0014** | **0.0045** |
| miR-181c-5p | -0.46 | **0.0354** | 0.0643 |
| miR-107 | -0.46 | **0.0205** | **0.0408** |
| let-7b-5p | -0.46 | **0.0485** | 0.0848 |
| miR-199a-3p | -0.50 | 0.1258 | 0.1853 |
| miR-199b-3p | -0.50 | 0.1257 | 0.1853 |
| miR-143-3p | -0.57 | **0.0469** | 0.0828 |
| miR-205-5p | -0.58 | 0.2065 | 0.2799 |
| miR-486-3p | -0.60 | **0.0400** | 0.0719 |
| miR-598-3p | -0.62 | **0.0054** | **0.0131** |
| miR-421 | -0.65 | **0.0003** | **0.0014** |
| miR-25-3p | -0.68 | **0.0039** | **0.0101** |
| miR-92a-3p | -0.70 | **0.0004** | **0.0015** |
| miR-30e-3p | -0.72 | **0.0082** | **0.0183** |
| miR-93-3p | -0.73 | **0.0003** | **0.0014** |
| miR-181a-3p | -0.74 | **0.0017** | **0.0053** |
| miR-98-5p | -0.74 | **0.0005** | **0.0017** |
| miR-155-5p | -0.74 | **0.0056** | **0.0133** |
| miR-181a-5p | -0.75 | **<0.0001** | **0.0001** |
| miR-486-5p | -0.75 | **0.0170** | **0.0346** |
| let-7g-5p | -0.75 | **0.0051** | **0.0126** |
| miR-26b-5p | -0.76 | **0.0008** | **0.0026** |
| miR-26a-5p | -0.77 | **0.0001** | **0.0003** |
| miR-375-3p | -0.78 | 0.1967 | 0.2704 |
| miR-584-5p | -0.81 | **0.0019** | **0.0056** |
| miR-16-2-3p | -0.84 | **<0.0001** | **0.0001** |
| miR-181d-5p | -0.87 | **0.0013** | **0.0043** |
| miR-382-5p | -0.89 | **0.0262** | 0.0517 |
| miR-409-3p | -0.91 | **0.0269** | 0.0523 |
| miR-125a-5p | -0.91 | **<0.0001** | **0.0002** |
| miR-146b-5p | -0.96 | **<0.0001** | **<0.0001** |
| let-7a-5p | -0.98 | **0.0005** | **0.0019** |
| miR-146a-5p | -1.00 | **<0.0001** | **0.0001** |
| miR-543 | -1.07 | **0.0133** | **0.0283** |
| miR-363-3p | -1.10 | **<0.0001** | **<0.0001** |
| miR-10a-5p | -1.13 | **<0.0001** | **<0.0001** |
| miR-487b-3p | -1.13 | **0.0152** | **0.0320** |
| miR-342-3p | -1.17 | **<0.0001** | **<0.0001** |
| miR-423-3p | -1.21 | **<0.0001** | **0.0001** |
| miR-183-5p | -1.27 | **0.0004** | **0.0015** |
| miR-145-3p | -1.28 | **<0.0001** | **0.0003** |
| miR-182-5p | -1.29 | **<0.0001** | **0.0002** |
| miR-10b-5p | -1.32 | **<0.0001** | **<0.0001** |
| miR-144-5p | -1.42 | **<0.0001** | **<0.0001** |
| let-7f-5p | -1.43 | **<0.0001** | **<0.0001** |
| miR-4508 | -1.69 | **<0.0001** | **0.0001** |
| miR-181a-2-3p | -1.75 | **<0.0001** | **<0.0001** |
| miR-4433b-5p | -1.81 | **0.0024** | **0.0068** |
| miR-150-5p | -2.18 | **<0.0001** | **<0.0001** |
| miR-215-5p | -3.94 | **<0.0001** | **<0.0001** |

Differential expression analysis of RNA-Seq data was performed using edgeR and applying the independent filtering method of DESeq2 to remove low abundant miRNA to optimize the Benjamini-Hochberg false discovery rate (FDR) correction.

**Table S4. List of miRNAs assessed by RNA-Seq in plasma of COVID-19 patients with non-severe (n = 18) and severe (n = 18) disease, ranked by logarithmic fold change (Log_2_-FC).**

| **miRNA** | **Log_2_-FC** | ***P* value** | **FDR** | ***P* value**  **(BMI adjusted)** | **FDR**  **(BMI adjusted)** |
| --- | --- | --- | --- | --- | --- |
| miR-10396b-5p | 2.95 | **0.0004** | **0.0036** | **0.0003** | **0.0052** |
| miR-10396a-5p | 2.95 | **0.0004** | **0.0036** | **0.0003** | **0.0052** |
| miR-122-5p | 2.82 | **0.0006** | **0.0045** | **0.0096** | **0.0357** |
| miR-193b-3p | 2.47 | **0.0001** | **0.0017** | **0.0007** | **0.0070** |
| miR-4454 | 2.40 | **0.0004** | **0.0036** | **0.0087** | **0.0332** |
| miR-122-3p | 2.10 | **0.0020** | **0.0088** | **0.0048** | **0.0209** |
| miR-133a-3p | 2.06 | **0.0016** | **0.0077** | **0.0128** | **0.0436** |
| miR-99a-5p | 1.92 | **0.0003** | **0.0032** | **0.0033** | **0.0177** |
| miR-3960 | 1.92 | **0.0006** | **0.0045** | **0.0046** | **0.0205** |
| miR-885-5p | 1.73 | **0.0021** | **0.0088** | **0.0039** | **0.0191** |
| miR-100-5p | 1.71 | **0.0010** | **0.0058** | **0.0049** | **0.0209** |
| miR-148a-3p | 1.69 | **0.0001** | **0.0017** | **0.0005** | **0.0061** |
| miR-192-5p | 1.63 | **0.0013** | **0.0071** | **0.0024** | **0.0147** |
| miR-199b-5p | 1.54 | **<0.0001** | **0.0006** | **<0.0001** | **0.0006** |
| miR-210-3p | 1.45 | **0.0001** | **0.0017** | **0.0003** | **0.0052** |
| miR-193a-5p | 1.45 | **0.0006** | **0.0045** | **0.0021** | **0.0133** |
| miR-27b-3p | 1.42 | **0.0008** | **0.0051** | **0.0040** | **0.0191** |
| miR-574-3p | 1.38 | **<0.0001** | **0.0012** | **0.0001** | **0.0028** |
| miR-378a-3p | 1.35 | **0.0001** | **0.0018** | **0.0004** | 0.0059 |
| miR-34a-5p | 1.27 | **0.0062** | **0.0214** | **0.0257** | 0.0759 |
| miR-125b-5p | 1.09 | **0.0048** | **0.0179** | **0.0204** | 0.0620 |
| miR-1290 | 1.07 | 0.0812 | 0.1690 | 0.2509 | 0.4120 |
| miR-30a-5p | 1.06 | **0.0007** | **0.0047** | **0.0036** | **0.0187** |
| miR-4488 | 0.96 | **0.0648** | 0.1415 | 0.0635 | 0.1516 |
| miR-141-3p | 0.93 | **0.0133** | **0.0407** | 0.1658 | 0.3059 |
| miR-505-3p | 0.93 | **0.0014** | **0.0071** | **0.0032** | **0.0177** |
| miR-941 | 0.85 | **0.0001** | **0.0017** | **0.0002** | **0.0052** |
| miR-22-3p | 0.84 | **0.0082** | **0.0276** | **0.0118** | **0.0414** |
| miR-1246 | 0.83 | 0.2169 | 0.3435 | 0.5911 | 0.7558 |
| miR-424-3p | 0.81 | **0.0001** | **0.0017** | **0.0005** | **0.0061** |
| miR-223-5p | 0.81 | **0.0007** | **0.0047** | **0.0031** | **0.0177** |
| miR-29a-3p | 0.81 | **0.0021** | **0.0088** | **0.0028** | **0.0167** |
| miR-152-3p | 0.81 | **0.0061** | **0.0214** | **0.0130** | **0.0436** |
| miR-375-3p | 0.77 | 0.1212 | 0.2311 | 0.1357 | 0.2729 |
| miR-483-5p | 0.72 | 0.1077 | 0.2119 | 0.1145 | 0.2355 |
| miR-130b-3p | 0.70 | **<0.0001** | **0.0008** | **<0.0001** | **0.0006** |
| miR-345-5p | 0.68 | **0.0093** | **0.0309** | **0.0140** | **0.0456** |
| miR-197-3p | 0.66 | **0.0031** | **0.0122** | **0.0087** | **0.0332** |
| miR-497-5p | 0.63 | **0.0384** | **0.0916** | 0.0639 | 0.1516 |
| miR-30a-3p | 0.59 | 0.1313 | 0.2449 | 0.3120 | 0.4693 |
| miR-200a-3p | 0.58 | 0.1213 | 0.2311 | 0.9229 | 0.9673 |
| miR-99b-5p | 0.56 | **0.0244** | **0.0641** | 0.0552 | 0.1372 |
| miR-7-1-3p | 0.56 | **0.0015** | **0.0073** | **0.0014** | **0.0103** |
| miR-29c-3p | 0.53 | **0.0110** | **0.0353** | **0.0113** | **0.0403** |
| miR-21-3p | 0.52 | 0.0619 | 0.1385 | 0.0683 | 0.1547 |
| miR-324-3p | 0.52 | **0.0038** | **0.0145** | **0.0053** | **0.0219** |
| miR-320b | 0.51 | 0.0723 | 0.1559 | 0.0797 | 0.1784 |
| miR-132-3p | 0.51 | **0.0189** | **0.0528** | **0.0472** | 0.1207 |
| miR-130a-3p | 0.50 | **0.0010** | **0.0058** | **0.0007** | **0.0070** |
| miR-139-5p | 0.50 | 0.0871 | 0.1752 | 0.1587 | 0.2991 |
| miR-425-3p | 0.49 | 0.0014 | **0.0071** | **0.0041** | **0.0191** |
| miR-23b-3p | 0.47 | 0.0585 | 0.1326 | 0.1372 | 0.2729 |
| miR-1307-3p | 0.46 | **0.0136** | **0.0407** | **0.0382** | 0.1086 |
| miR-223-3p | 0.42 | 0.0870 | 0.1752 | 0.0917 | 0.1978 |
| let-7a-3p | 0.41 | 0.0238 | **0.0635** | **0.0491** | 0.1239 |
| miR-17-3p | 0.37 | **0.0371** | **0.0897** | 0.0662 | 0.1519 |
| miR-24-3p | 0.37 | 0.0641 | 0.1415 | 0.0631 | 0.1516 |
| miR-30d-5p | 0.37 | 0.0901 | 0.1792 | 0.1882 | 0.3335 |
| miR-21-5p | 0.34 | 0.1259 | 0.2373 | 0.1957 | 0.3401 |
| miR-92b-3p | 0.33 | 0.1641 | 0.2825 | 0.3881 | 0.5428 |
| miR-33a-5p | 0.32 | **0.0352** | **0.0864** | **0.0465** | 0.1206 |
| miR-30e-5p | 0.31 | 0.2447 | 0.3776 | 0.3293 | 0.4869 |
| miR-151b | 0.31 | 0.1406 | 0.2541 | 0.1465 | 0.2851 |
| miR-28-3p | 0.28 | 0.1333 | 0.2460 | 0.1525 | 0.2936 |
| miR-199a-5p | 0.26 | 0.3165 | 0.4644 | 0.2381 | 0.3983 |
| miR-660-5p | 0.26 | 0.1733 | 0.2955 | 0.1712 | 0.3103 |
| miR-128-3p | 0.25 | **0.0261** | 0.0677 | **0.0395** | 0.1105 |
| miR-323b-3p | 0.24 | 0.5604 | 0.6871 | 0.2579 | 0.4181 |
| miR-145-5p | 0.24 | 0.2965 | 0.4439 | 0.3725 | 0.5250 |
| miR-27a-3p | 0.20 | 0.1181 | 0.2297 | 0.1569 | 0.2988 |
| miR-484 | 0.20 | 0.2511 | 0.3842 | 0.1864 | 0.3335 |
| miR-532-5p | 0.19 | 0.2068 | 0.3334 | 0.2153 | 0.3636 |
| miR-361-5p | 0.17 | 0.1526 | 0.2678 | 0.2792 | 0.4435 |
| let-7d-3p | 0.16 | 0.4742 | 0.6195 | 0.6664 | 0.8170 |
| miR-425-5p | 0.15 | 0.2248 | 0.3530 | 0.2075 | 0.3537 |
| miR-3615 | 0.15 | 0.3890 | 0.5316 | 0.5225 | 0.6878 |
| miR-324-5p | 0.12 | 0.3489 | 0.4956 | 0.3062 | 0.4693 |
| miR-17-5p | 0.12 | 0.5350 | 0.6651 | 0.4998 | 0.6627 |
| miR-221-3p | 0.12 | 0.5174 | 0.6591 | 0.7253 | 0.8541 |
| miR-142-3p | 0.12 | 0.5275 | 0.6649 | 0.5507 | 0.7195 |
| miR-30c-5p | 0.11 | 0.5927 | 0.7120 | 0.6180 | 0.7739 |
| miR-338-3p | 0.11 | 0.5321 | 0.6651 | 0.9328 | 0.9708 |
| miR-29b-3p | 0.08 | 0.7033 | 0.8018 | 0.6183 | 0.7739 |
| miR-151a-3p | 0.08 | 0.6153 | 0.7295 | 0.7837 | 0.8768 |
| miR-15b-5p | 0.07 | 0.7290 | 0.8152 | 0.5637 | 0.7282 |
| miR-143-3p | 0.07 | 0.7941 | 0.8667 | 0.9832 | 0.9896 |
| miR-361-3p | 0.06 | 0.6657 | 0.7688 | 0.7757 | 0.8733 |
| miR-222-3p | 0.05 | 0.7332 | 0.8152 | 0.9399 | 0.9725 |
| miR-532-3p | 0.04 | 0.8644 | 0.9167 | 0.7637 | 0.8652 |
| miR-199b-3p | 0.03 | 0.9003 | 0.9325 | 0.7199 | 0.8540 |
| miR-199a-3p | 0.03 | 0.9010 | 0.9325 | 0.7204 | 0.8540 |
| miR-30b-5p | 0.03 | 0.9012 | 0.9325 | 0.9458 | 0.9730 |
| miR-22-5p | 0.03 | 0.9149 | 0.9412 | 0.8712 | 0.9451 |
| miR-148b-3p | 0.01 | 0.9511 | 0.9708 | 0.7612 | 0.8652 |
| miR-151a-5p | 0.01 | 0.9790 | 0.9845 | 0.8951 | 0.9538 |
| miR-15a-5p | 0.00 | 0.9930 | 0.9930 | 0.7170 | 0.8540 |
| miR-501-3p | -0.01 | 0.9654 | 0.9763 | 0.8602 | 0.9389 |
| miR-500a-3p | -0.01 | 0.9545 | 0.9708 | 0.9241 | 0.9673 |
| miR-28-5p | -0.03 | 0.8875 | 0.9325 | 0.9513 | 0.9730 |
| miR-191-5p | -0.03 | 0.8614 | 0.9167 | 0.8267 | 0.9134 |
| miR-140-3p | -0.03 | 0.7733 | 0.8545 | 0.7631 | 0.8652 |
| miR-125a-5p | -0.05 | 0.7878 | 0.8651 | 0.3566 | 0.5106 |
| miR-93-5p | -0.05 | 0.7301 | 0.8152 | 0.8792 | 0.9481 |
| miR-502-3p | -0.05 | 0.8108 | 0.8796 | 0.8323 | 0.9140 |
| miR-421 | -0.06 | 0.6795 | 0.7797 | 0.6639 | 0.8170 |
| miR-103a-3p | -0.06 | 0.6306 | 0.7380 | 0.9999 | 0.9999 |
| miR-376c-3p | -0.07 | 0.8655 | 0.9167 | 0.8849 | 0.9485 |
| miR-654-3p | -0.07 | 0.8546 | 0.9167 | 0.9582 | 0.9746 |
| miR-423-5p | -0.07 | 0.6376 | 0.7411 | 0.7626 | 0.8652 |
| miR-106b-3p | -0.07 | 0.5831 | 0.7053 | 0.6981 | 0.8443 |
| miR-32-5p | -0.08 | 0.7158 | 0.8109 | 0.9841 | 0.9896 |
| miR-107 | -0.09 | 0.6027 | 0.7192 | 0.9167 | 0.9673 |
| miR-598-3p | -0.10 | 0.5192 | 0.6591 | 0.6641 | 0.8170 |
| miR-101-3p | -0.11 | 0.5586 | 0.6871 | 0.6967 | 0.8443 |
| miR-181a-3p | -0.12 | 0.5190 | 0.6591 | 0.3610 | 0.5128 |
| miR-186-5p | -0.12 | 0.3997 | 0.5420 | 0.4718 | 0.6398 |
| miR-142-5p | -0.13 | 0.3217 | 0.4644 | 0.4974 | 0.6627 |
| miR-301a-3p | -0.13 | 0.4889 | 0.6341 | 0.7414 | 0.8652 |
| miR-23a-3p | -0.14 | 0.4275 | 0.5641 | 0.3293 | 0.4869 |
| miR-127-3p | -0.16 | 0.6308 | 0.7380 | 0.7889 | 0.8771 |
| let-7i-5p | -0.17 | 0.2159 | 0.3435 | 0.1907 | 0.3346 |
| miR-25-3p | -0.18 | 0.3568 | 0.5029 | 0.3546 | 0.5106 |
| miR-10a-5p | -0.18 | 0.4073 | 0.5482 | 0.1321 | 0.2687 |
| miR-16-5p | -0.18 | 0.3660 | 0.5118 | 0.5973 | 0.7583 |
| miR-196b-5p | -0.18 | 0.3759 | 0.5216 | 0.3102 | 0.4693 |
| miR-185-5p | -0.19 | 0.4180 | 0.5584 | 0.5655 | 0.7282 |
| miR-92a-3p | -0.19 | 0.2025 | 0.3295 | 0.2800 | 0.4435 |
| miR-181b-5p | -0.20 | 0.2749 | 0.4171 | 0.1131 | 0.2354 |
| miR-423-3p | -0.21 | 0.2976 | 0.4439 | 0.3382 | 0.4922 |
| miR-106b-5p | -0.21 | 0.3257 | 0.4664 | 0.3918 | 0.5437 |
| miR-625-3p | -0.21 | 0.4286 | 0.5641 | 0.3318 | 0.4869 |
| miR-652-3p | -0.23 | 0.0780 | 0.1643 | 0.1069 | 0.2278 |
| miR-335-5p | -0.23 | 0.3804 | 0.5238 | 0.4869 | 0.6553 |
| miR-320a-3p | -0.23 | 0.3138 | 0.4642 | 0.4509 | 0.6161 |
| miR-376a-3p | -0.24 | 0.5828 | 0.7053 | 0.7489 | 0.8652 |
| miR-20b-5p | -0.25 | 0.3205 | 0.4644 | 0.4375 | 0.6024 |
| miR-744-5p | -0.27 | 0.1787 | 0.2997 | 0.3110 | 0.4693 |
| miR-30e-3p | -0.28 | 0.1813 | 0.3005 | 0.1716 | 0.3103 |
| miR-126-5p | -0.29 | 0.0827 | 0.1702 | 0.0837 | 0.1850 |
| miR-20a-5p | -0.29 | 0.1414 | 0.2541 | 0.2493 | 0.4120 |
| miR-486-3p | -0.34 | 0.1941 | 0.3187 | 0.2592 | 0.4181 |
| miR-146a-5p | -0.37 | **0.0427** | 0.1005 | **0.0417** | 0.1148 |
| miR-181a-5p | -0.37 | **0.0133** | **0.0407** | **0.0107** | **0.0390** |
| miR-126-3p | -0.39 | **0.0178** | **0.0514** | **0.0132** | **0.0436** |
| miR-145-3p | -0.40 | 0.1475 | 0.2613 | 0.0644 | 0.1516 |
| miR-382-5p | -0.42 | 0.2339 | 0.3641 | 0.3026 | 0.4693 |
| let-7g-5p | -0.43 | **0.0344** | 0.0855 | 0.0657 | 0.1519 |
| miR-224-5p | -0.43 | 0.1590 | 0.2763 | 0.1637 | 0.3052 |
| miR-144-3p | -0.44 | **0.0561** | 0.1288 | 0.1126 | 0.2354 |
| miR-15b-3p | -0.44 | **0.0734** | 0.1565 | 0.1417 | 0.2788 |
| miR-146b-5p | -0.46 | **0.0027** | **0.0108** | **0.0009** | **0.0082** |
| miR-19b-3p | -0.48 | **0.0053** | **0.0191** | **0.0170** | 0.0534 |
| miR-454-3p | -0.48 | **0.0183** | **0.0520** | **0.0462** | 0.1206 |
| miR-409-3p | -0.49 | 0.1791 | 0.2997 | 0.2961 | 0.4649 |
| miR-19a-3p | -0.50 | **0.0021** | **0.0088** | **0.0080** | **0.0319** |
| miR-181d-5p | -0.52 | **0.0201** | **0.0552** | **0.0163** | 0.0520 |
| miR-98-5p | -0.53 | **0.0018** | **0.0082** | **0.0016** | **0.0108** |
| miR-10b-5p | -0.54 | **0.0300** | **0.0757** | **0.0074** | **0.0301** |
| let-7b-5p | -0.54 | **0.0215** | **0.0582** | **0.0259** | 0.0759 |
| miR-486-5p | -0.54 | 0.0549 | 0.1276 | 0.0898 | 0.1960 |
| miR-340-5p | -0.62 | **0.0102** | **0.0333** | **0.0277** | 0.0799 |
| miR-451a | -0.64 | **0.0177** | **0.0514** | **0.0446** | 0.1206 |
| miR-487b-3p | -0.64 | 0.1420 | 0.2541 | 0.2047 | 0.3523 |
| miR-16-2-3p | -0.70 | **0.0005** | **0.0041** | **0.0011** | **0.0094** |
| miR-181c-5p | -0.70 | **0.0014** | **0.0071** | **0.0036** | **0.0187** |
| miR-26a-5p | -0.73 | **0.0002** | **0.0021** | **0.0003** | **0.0054** |
| let-7f-5p | -0.73 | **0.0003** | **0.0032** | **0.0007** | **0.0070** |
| miR-342-3p | -0.75 | **0.0013** | **0.0070** | **0.0013** | **0.0101** |
| miR-183-5p | -0.76 | **0.0051** | **0.0187** | **0.0018** | **0.0116** |
| miR-26b-5p | -0.76 | **0.0002** | **0.0029** | **0.0007** | **0.0070** |
| miR-181a-2-3p | -0.79 | **0.0007** | **0.0047** | **0.0012** | **0.0096** |
| miR-150-5p | -0.85 | **0.0022** | **0.0088** | **0.0044** | **0.0200** |
| miR-584-5p | -0.86 | **0.0001** | **0.0017** | **0.0003** | **0.0052** |
| let-7a-5p | -0.87 | **0.0007** | **0.0047** | **0.0012** | **0.0096** |
| miR-363-3p | -0.87 | **<0.0001** | **0.0010** | **0.0001** | **0.0028** |
| miR-144-5p | -0.94 | **0.0001** | **0.0017** | **0.0005** | **0.0064** |
| miR-543 | -1.08 | **0.0135** | **0.0407** | **0.0193** | 0.0595 |
| miR-369-3p | -1.08 | **0.0270** | **0.0691** | **0.0464** | 0.1206 |
| miR-182-5p | -1.13 | **<0.0001** | **0.0006** | **<0.0001** | **0.0006** |

Differential expression analysis of RNA-Seq data was performed using edgeR and applying the independent filtering method of DESeq2 to remove low abundant miRNA to optimize the Benjamini-Hochberg false discovery rate (FDR) correction.

**Table S5. Clinical characteristics of COVID-19 patients for whom baseline plasma samples were used for RT-qPCR.**

| **Clinical Characteristics** | **Mild (*n*=6)** | **Moderate (*n*=39)** | **Severe (*n*=16)** | ***P* value** |
| --- | --- | --- | --- | --- |
| SARS-CoV-2 RNAemia within first 6 ICU days (%) | 0.0 | 5.3 | 53.9 | **0.0001** |
| Non-Survivors 28 days after hospitalization (%) | 0.0 | 13.2 | 30.8 | 0.17 |
| **Demographics** |  |  |  |  |
| Age (years) | 60.5 (40.5, 80.5) | 60.5 (35.5, 85.5) | 62.0 (47.5, 76.5) | 0.99 |
| Sex (% male) | 16.7 | 73.7 | 84.6 | **0.008** |
| BMI (kg/m^2^) | 24.5 (19.5, 29.5) | 27.0 (22.0, 32.0) | 28.2 (22.3, 34.1) | 0.16 |
| **Pre-existing conditions** |  |  |  |  |
| Cancer, active (%) | 0.0 | 18.0 | 0.0 | 0.11 |
| COPD (%) | 0.0 | 21.1 | 7.7 | 0.28 |
| Diabetes (%) | 16.7 | 29.0 | 31.0 | 0.80 |
| Heart disease, congestive (%) | 16.7 | 5.1 | 6.25 | 0.57 |
| Heart disease, ischemic (%) | 50.0 | 15.4 | 25.0 | 0.05 |
| Hypercholesterinaemia (%) | 16.7 | 10.3 | 31.3 | 0.16 |
| Hypertension (%) | 66.7 | 42.1 | 46.2 | 0.53 |
| Smokers (%) | 33.3 | 33.3 | 18.8 | 0.55 |
| **Therapy during inpatient care** |  |  |  |  |
| Antivirals (%) | 0.0 | 5.1 | 37.5 | **0.003** |
| Corticosteroids (%) | 16.7 | 15.4 | 43.8 | 0.07 |
| Vasopressors (%) | 0.0 | 0.0 | 68.8 | **<0.0001** |
| **Acute Care Parameters** |  |  |  |  |
| BP systolic (mmHg) | 123 (120, 125) | 125 (117, 147) | 107 (102, 140) | 0.09 |
| BP diastolic (mmHg) | 84 (80, 87) | 78 (70, 88) | 60 (55, 72) | **0.001** |
| MAP (mmHg) | - | - | 74 (68, 83) |  |
| FiO_2_ (fraction of 1) | 0.21 (0.21, 0.21) | 0.28 (0.21, 0.28) | 0.50 (0.45, 0.60) | **<0.0001** |
| Heart rate (bpm) | 107 (104, 110) | 95 (85, 119) | 95 (90, 114) | 0.85 |
| Temperature (°C) | 37.6 (37.6, 37.6) | 37.5 (36.8, 38.3) | 38.1 (37.3, 38.8) | 0.27 |
| **Blood Biochemistry** |  |  |  |  |
| Albumin (g/L) | 40.0 (37.0, 43.0) | 37.0 (32.0, 39.0) | 30.0 (27.0, 31.0) | **<0.0001** |
| ALT (IU/L) | 13.0 (7.0, 14.0) | 33.0 (19.0, 55.0) | 31.0 (18.0, 107.0) | **0.0329** |
| Bilirubin (µmol/L) | 6.0 (4.0, 6.0) | 8.0 (4.8, 11.0) | 6.0 (5.5, 14.0) | 0.38 |
| Creatinine (µmol/L) | 308.0 (40.0, 652.0) | 71.0 (63.0, 88.0) | 81.0 (60.0, 139.0) | 0.71 |
| C-reactive protein (mg/L) | 16.0 (2.3, 30.0) | 60.0 (30.0, 140.0) | 234.0 (158.0, 338.0) | **<0.0001** |
| D-dimer (ng/ml) | - | 0.62 (0.44, 1.31) | 3.97 (2.25, 7.78) | **0.0005** |
| Ferritin (µg/L) | 564 (107, 1654) | 889 (512, 1812) | 976 (497, 1829) | 0.63 |
| Glucose (mmol/L) | 4.80 (4.80, 4.80) | 7.43 (6.21, 9.25) | 8.40 (6.70, 11.60) | 0.15 |
| Phosphate (mmol/L) | 1.15 (0.60, 1.63) | 0.90 (0.80, 1.10) | 0.91 (0.81, 1.06) | 0.81 |
| Troponin T (ng/L) | - | 8.5 (5.0, 18.8) | 18.5 (11.5, 26.8) | 0.12 |
| **Blood Count** |  |  |  |  |
| Lymphocytes (10^9^/l) | 1.20 (0.75, 1.90) | 1.00 (0.60, 1.30) | 1.00 (0.87, 2.00) | 0.30 |
| Monocytes (10^9^/l) | 0.40 (0.25, 0.70) | 0.50 (0.40, 0.70) | 0.29 (0.21, 0.45) | **0.0089** |
| Neutrophils (10^9^/l) | 3.70 (2.20, 6.10) | 5.50 (3.40, 8.10) | 9.40 (7.70, 11.00) | **0.005** |
| Platelets (10^9^/l) | 259 (204, 389) | 264 (191, 338) | 313 (198, 392) | 0.92 |
| White cell count (10^9^/l) | 5.90 (3.60, 8.30) | 7.50 (4.80, 9.30) | 12.00 (9.50, 13.00) | **0.0078** |

Abbreviations: ALT: alanine aminotransferase, BMI: body mass index, BP: blood pressure, COPD: chronic obstructive pulmonary disease, FiO_2_: fraction of inspired oxygen, MAP: mean arterial blood pressure. For continuous variables, the medians and interquartile ranges are shown, and Kruskal-Wallis tests were used for statistical comparisons. For binary variables, percentages are shown, and Chi-square tests were used for statistical comparisons.

**Table S6. Plasma levels of stably detectable (>70%) miRNAs in COVID-19 patients with mild (*n* = 6), moderate (*n* = 39) and severe (*n* = 16) COVID-19.**

| **Target** | **Fold change (Log_2_)** | | |  | **FDR** | | |  |
| --- | --- | --- | --- | --- | --- | --- | --- | --- |
|  | **Mild**  **versus**  **moderate** | **Moderate**  **versus**  **severe** | **Mild**  **versus**  **severe** |  | **Unadjusted** | **Adjusted for**  **age, sex** | **Adjusted for age, sex, BMI** | |
| miR-133a | 1.10 | 1.74 | 2.84 |  | **0.0008** | **0.003** | **0.005** | |
| miR-122 | 2.18 | 1.48 | 3.66 |  | **0.0008** | **0.038** | **0.077** | |
| miR-126 | 1.29 | 1.29 | 2.57 |  | **0.0008** | **0.017** | **0.026** | |
| miR-21 | 1.12 | 0.99 | 2.11 |  | **0.0008** | 0.16 | 0.26 | |
| miR-197 | 1.30 | 1.29 | 2.60 |  | **0.0012** | 0.08 | 0.14 | |
| miR-320a | 0.52 | 0.96 | 1.48 |  | **0.0049** | 0.15 | 0.27 | |
| miR-223 | 1.93 | 0.31 | 2.24 |  | **0.0246** | 0.43 | 0.52 | |
| miR-210 | 0.44 | 1.18 | 1.62 |  | **0.0266** | 0.11 | 0.23 | |
| miR-192 | 1.42 | 0.15 | 1.58 |  | **0.0341** | 0.30 | 0.54 | |
| miR-181b | -0.01 | 0.56 | 0.55 |  | 0.06 | 0.08 | 0.93 | |
| miR-150 | 1.16 | 0.00 | 1.16 |  | 0.67 | 0.81 | 0.80 | |

Significance between the three severity groups was determined using ANOVA on logarithmised relative quantity (log_2_-RQ) values, then applying Benjamini and Hochberg’s FDR correction for the 14 miRNA targets.

**Table S7. Plasma detectability of miRNAs with overall detectability <70% in COVID-19 patients with different disease severities.**

| **Target** | **Mild**  ***n* = 6**  **% detect.** | **Moderate**  ***n* = 39**  **% detect.** | **Severe**  ***n* = 16**  **% detect.** | **FDR** | | |
| --- | --- | --- | --- | --- | --- | --- |
|  |  |  |  | **Unadjusted** | **Adjusted for**  **age, sex** | **Adjusted for**  **age, sex, BMI** |
| miR-208b | 0 | 0 | 38 | **0.004** | **0.004** | **0.004** |
| miR-187 | 0 | 0 | 31 | **0.005** | **0.009** | **0.009** |
| miR-124 | 17 | 34 | 38 | 0.47 | 0.47 | 0.57 |

Significance between the three severity groups was determined using Chi-square tests, applying Benjamini and Hochberg’s FDR correction for the 14 miRNA targets.

**Table S8. Clinical characteristics of COVID-19 ICU patients for whom baseline serum samples were available (n = 65).**

| **Clinical Characteristics** | **ICU patients (*n*=65)** | **Survivors (*n*=48)** | **Non-survivors (*n*=17)** | ***P* value** |
| --- | --- | --- | --- | --- |
| SARS-COV-2 RNAemia within first 6 ICU days (%) | 24.6 | 12.5 | 58.8 | **0.0001** |
| Days POS until ICU admission | 7.0 (7.0, 11.0) | 7.0 (6.0, 10.0) | 10.0 (7.0, 14.0) | 0.20 |
| Days POS until death | 22.0 (20.0, 38.0) | - | 22.0 (19.0, 27.0) | - |
| Days from admission to death | 14.0 (11.0, 18.0) | - | 13.0 (11.0, 15.0) | - |
| **Demographics** |  |  |  |  |
| Age (years) | 54.0 (45.0, 64.0) | 52.0 (44.0, 61.0) | 66.0 (55.0, 79.0) | **0.0014** |
| Sex (% male) | 71.4 | 70.8 | 73.3 | 0.85 |
| BMI (kg/m^2^) | 28.0 (25.0, 34.0) | 28.0 (25.0, 35.0) | 26.0 (25.0, 32.0) | 0.23 |
| **Pre-existing conditions** |  |  |  |  |
| COPD (%) | 15.9 | 14.6 | 20.0 | 0.62 |
| Diabetes (%) | 28.6 | 29.2 | 26.7 | 0.85 |
| Hypertension (%) | 38.1 | 33.3 | 53.3 | 0.16 |
| Liver cirrhosis (%) | 1.6 | 2.1 | 0 | 0.57 |
| Myocardial infarction (%) | 3.2 | 2.1 | 6.7 | 0.38 |
| Renal disease (%) | 6.3 | 8.3 | 0 | 0.25 |
| **Pre-medication** |  |  |  |  |
| Antidiabetic (%) | 38.5 | 33.3 | 52.9 | 0.15 |
| Antihypertensive (%) | 46.0 | 38.3 | 68.8 | **0.038** |
| **Therapy during inpatient care** |  |  |  |  |
| Antivirals (%) | 9.2 | 8.3 | 11.8 | 0.68 |
| Corticosteroids (%) | 66.2 | 62.5 | 76.5 | 0.30 |
| Vasopressors (%) | 75.4 | 68.8 | 94.1 | **0.0369** |
| **Acute Care Parameters** |  |  |  |  |
| FiO_2_ (fraction of 1) | 0.50 (0.35, 0.60) | 0.45 (0.35, 0.54) | 0.50 (0.45, 0.60) | 0.07 |
| Heart rate (bpm) | 96 (64, 111) | 98 (63, 115) | 92 (80, 101) | 0.49 |
| MAP (mmHg) | 65 (60, 80) | 64 (59, 83) | 65 (59, 70) | 0.69 |
| Respiratory rate (bpm) | 22 (17, 30) | 22 (16, 29) | 23 (20, 32) | 0.50 |
| Temperature (°C) | 39 (37, 39) | 39 (37, 39) | 38 (37, 39) | 0.47 |
| **Blood Biochemistry** |  |  |  |  |
| Albumin (g/L) | 30.0 (27.0, 33.0) | 31.0 (28.0, 34.0) | 30.0 (26.0, 30.0) | 0.05 |
| ALP (U/L) | 62.0 (49.0, 85.0) | 62.0 (48.0, 91.0) | 61.0 (51.0, 78.0) | 0.77 |
| ALT (IU/L) | 32.0 (23.0, 54.0) | 32.0 (22.0, 56.0) | 30.0 (26.0, 54.0) | 0.76 |
| Anti-SARS-CoV-2 IgG ratio | 1.23 (0.38, 8.09) | 1.62 (0.46, 7.84) | 0.54 (0.25, 9.30) | 0.33 |
| Anti-SARS-CoV-2 neutralization (%) | 6.89 (0.00, 55.66) | 7.80 (0.00, 57.03) | 6.40 (0.00, 55.59 | 0.67 |
| Bilirubin (µmol/L) | 9.0 (6.0, 15.0) | 10.0 (6.0, 14.0) | 8.00 (6.80, 17.0) | 0.86 |
| NT-pro-BNP (pg/mL) | 228.0 (56.0, 706.0) | 105.0 (56.0, 706.0) | 544.0 (150.8, 1346.5) | 0.37 |
| Creatinine (µmol/L) | 92.0 (68.0, 166.0) | 85.0 (66.0, 164.0) | 132.0 (81.0, 174.0) | 0.23 |
| CK (U/L) | 223.0 (78.3, 519.5) | 179.0 (78.0, 382.0) | 529.0 (110.5, 1398.5) | 0.13 |
| C-reactive protein (mg/L) | 234.0 (136.0, 325.0) | 238.0 (98.0, 335.0) | 217.0 (172.0, 316.0) | 0.70 |
| D-dimer (µg/ml) | 2.11 (0.98, 4.77) | 1.53 (0.83, 4.02) | 5.45 (1.10, 8.87) | 0.11 |
| Haemoglobin (g/L) | 117.0 (104.0, 125.0) | 117.0 (102.0, 126.0) | 119.0 (104.0, 125.0) | 0.87 |
| pH | 7.4 (7.3, 7.4) | 7.4 (7.3, 7.4) | 7.4 (7.3, 7.4) | 0.95 |
| Potassium (mmol/L) | 4.5 (4.3, 4.8) | 4.5 (4.3, 4.8) | 4.40 (4.2, 4.9) | 0.98 |
| Sodium (mmol/L) | 140.0 (138.0, 143.0) | 140.0 (137.0, 143.0) | 141.0 (138.0, 145.0) | 0.25 |
| Troponin T (ng/L) | 12.0 (8.0, 23.0) | 10.5 (7.3, 18.0) | 23.0 (11.0, 48.0) | **0.0119** |
| Urea (mmol/L) | 7.00 (5.0, 12.0) | 6.4 (4.8, 9.6) | 11.0 (5.4, 17.0) | **0.0444** |
| **Blood Count** |  |  |  |  |
| Haematocrit (%) | 38.0 (33.0, 41.0) | 37.0 (31.0, 41.0) | 39.0 (35.0, 42.0) | 0.24 |
| Lymphocytes (10^9^/l) | 0.90 (0.68, 1.20) | 0.90 (0.70, 1.40) | 0.87 (0.40, 1.20) | 0.32 |
| Monocytes (10^9^/l) | 0.40 (0.23, 0.57) | 0.40 (0.28, 0.56) | 0.40 (0.20, 0.60) | 0.78 |
| Neutrophils (10^9^/l) | 7.50 (4.60, 9.80) | 7.30 (4.70, 9.60) | 7.60 (3.80, 9.80) | 0.90 |
| White cell count (10^9^/l) | 8.80 (6.40, 12.00) | 9.10 (6.50, 11.00) | 8.60 (5.80, 12.00) | 0.86 |

Abbreviations: days POS: days post onset of symptoms, BMI: body mass index, CK: creatine kinase, COPD: chronic obstructive pulmonary disease, FiO_2_: fraction of inspired oxygen, ALP: alkaline phosphatase, ALT: alanine aminotransferase. Median and interquartile ranges are shown for continuous variables. Binary variables are shown as percentages. Significance was determined using Mann-Whitney tests for continuous variables and using Chi-square tests for binary variables.

**Table S9. Correlation of serum miR-133a levels with serum proteins in all measurements ranked based on *P* values (*n* = 240 samples from *n* = 62 COVID-19 ICU patients).**

| **Protein** | **Spearman *r*** | ***P* value** | **FDR** |
| --- | --- | --- | --- |
| MPO | 0.21 | **0.0012** | 0.1300 |
| F2 | 0.20 | **0.0018** | 0.1300 |
| PFN1 | 0.20 | **0.0024** | 0.1300 |
| MMP9 | 0.19 | **0.0025** | 0.1300 |
| APOL1 | 0.18 | **0.0063** | 0.2621 |
| GPX3 | 0.17 | **0.0087** | 0.3016 |
| IGHA1 | 0.16 | **0.0107** | 0.3179 |
| MASP2 | 0.16 | **0.0148** | 0.3467 |
| C8A | 0.16 | **0.0150** | 0.3467 |
| ECM1 | 0.15 | **0.0212** | 0.3903 |
| GP1BA | 0.15 | **0.0215** | 0.3903 |
| PROZ | 0.14 | **0.0266** | 0.3903 |
| PRG4 | 0.14 | **0.0272** | 0.3903 |
| APOM | 0.14 | **0.0282** | 0.3903 |
| SERPING1 | 0.14 | **0.0291** | 0.3903 |
| SFTPB | -0.14 | **0.0313** | 0.3903 |
| CFH | 0.14 | **0.0319** | 0.3903 |
| A1BG | 0.14 | **0.0355** | 0.4102 |
| CFD | -0.13 | **0.0426** | 0.4664 |
| IGFALS | 0.13 | **0.0464** | 0.4814 |
| TGFBI | -0.13 | **0.0486** | 0.4814 |
| IGD | 0.13 | 0.0525 | 0.4876 |
| HABP2 | 0.12 | 0.0559 | 0.4876 |
| C4A | 0.12 | 0.0585 | 0.4876 |
| CDH5 | -0.12 | 0.0586 | 0.4876 |
| BTD | 0.12 | 0.0625 | 0.5000 |
| IGHM | -0.12 | 0.0681 | 0.5054 |
| IGFBP4 | -0.12 | 0.0708 | 0.5054 |
| BCHE | 0.12 | 0.0720 | 0.5054 |
| IGHG2 | 0.12 | 0.0729 | 0.5054 |
| PLG | 0.11 | 0.0794 | 0.5161 |
| SHBG | -0.11 | 0.0859 | 0.5169 |
| PRSS3 | -0.11 | 0.0925 | 0.5169 |
| C5 | 0.11 | 0.0939 | 0.5169 |
| S100A9 | 0.11 | 0.0951 | 0.5169 |
| S100A8 | 0.11 | 0.0960 | 0.5169 |
| SERPINA6 | 0.11 | 0.0962 | 0.5169 |
| IGFBP3 | 0.11 | 0.0973 | 0.5169 |
| B2M | -0.11 | 0.0994 | 0.5169 |
| THBS1 | 0.10 | 0.1105 | 0.5517 |
| F11 | 0.10 | 0.1142 | 0.5517 |
| CD5L | -0.10 | 0.1180 | 0.5517 |
| C8B | 0.10 | 0.1199 | 0.5517 |
| APOF | 0.10 | 0.1205 | 0.5517 |
| PIGR | 0.10 | 0.1220 | 0.5517 |
| APOC4 | 0.10 | 0.1248 | 0.5523 |
| KNG1 | 0.10 | 0.1339 | 0.5678 |
| HBD | 0.10 | 0.1357 | 0.5678 |
| PON1 | 0.10 | 0.1365 | 0.5678 |
| ITIH2 | 0.10 | 0.1393 | 0.5681 |
| C1R | 0.09 | 0.1458 | 0.5832 |
| IGFBP2 | -0.09 | 0.1535 | 0.6024 |
| CPB2 | 0.09 | 0.1570 | 0.6032 |
| HRG | 0.09 | 0.1595 | 0.6032 |
| PCSK9 | 0.09 | 0.1650 | 0.6129 |
| NID1 | -0.09 | 0.1681 | 0.6134 |
| LCN2 | 0.09 | 0.1738 | 0.6201 |
| F9 | 0.09 | 0.1759 | 0.6201 |
| ITIH1 | 0.09 | 0.1818 | 0.6237 |
| C7 | 0.09 | 0.1829 | 0.6237 |
| HPR | 0.08 | 0.1963 | 0.6586 |
| IGKC | -0.08 | 0.2050 | 0.6704 |
| PPBP | 0.08 | 0.2095 | 0.6704 |
| CPN1 | 0.08 | 0.2166 | 0.6737 |
| PLXDC2 | 0.08 | 0.2170 | 0.6737 |
| CLU | 0.08 | 0.2265 | 0.6854 |
| IGFBP6 | -0.08 | 0.2304 | 0.6854 |
| CST3 | -0.08 | 0.2311 | 0.6854 |
| SPARC | 0.08 | 0.2345 | 0.6854 |
| GP5 | 0.07 | 0.2501 | 0.6854 |
| IGM | -0.07 | 0.2586 | 0.6854 |
| FGB | -0.07 | 0.2607 | 0.6854 |
| TTR | 0.07 | 0.2612 | 0.6854 |
| CFB | 0.07 | 0.2642 | 0.6854 |
| LCAT | 0.07 | 0.2650 | 0.6854 |
| DBH | -0.07 | 0.2663 | 0.6854 |
| APOE | 0.07 | 0.2681 | 0.6854 |
| AFM | 0.07 | 0.2704 | 0.6854 |
| TF | 0.07 | 0.2710 | 0.6854 |
| HBA1 | 0.07 | 0.2728 | 0.6854 |
| CFI | 0.07 | 0.2735 | 0.6854 |
| APOB | 0.07 | 0.2805 | 0.6946 |
| ALB | 0.07 | 0.2876 | 0.6946 |
| LUM | -0.07 | 0.2878 | 0.6946 |
| C8G | 0.07 | 0.2933 | 0.6946 |
| SERPINA1 | 0.07 | 0.2962 | 0.6946 |
| SERPINA10 | 0.07 | 0.2972 | 0.6946 |
| CLEC3B | 0.07 | 0.3035 | 0.6951 |
| APCS | 0.07 | 0.3041 | 0.6951 |
| SERPINC1 | 0.07 | 0.3106 | 0.7022 |
| APOC1 | 0.06 | 0.3177 | 0.7078 |
| F5 | 0.06 | 0.3240 | 0.7078 |
| APOD | 0.06 | 0.3256 | 0.7078 |
| CTSD | -0.06 | 0.3314 | 0.7078 |
| VTN | 0.06 | 0.3315 | 0.7078 |
| ITIH3 | 0.06 | 0.3335 | 0.7078 |
| CFHR5 | 0.06 | 0.3383 | 0.7108 |
| HBB | 0.06 | 0.3437 | 0.7139 |
| HPX | 0.06 | 0.3492 | 0.7139 |
| APOA4 | -0.06 | 0.3513 | 0.7139 |
| GLIPR2 | -0.06 | 0.3563 | 0.7139 |
| CFP | 0.06 | 0.3577 | 0.7139 |
| CPN2 | 0.06 | 0.3624 | 0.7139 |
| CHL1 | -0.06 | 0.3638 | 0.7139 |
| C1RL | 0.06 | 0.3724 | 0.7239 |
| SAA4 | 0.06 | 0.3767 | 0.7240 |
| A2M | -0.06 | 0.3822 | 0.7240 |
| FN1 | 0.06 | 0.3829 | 0.7240 |
| C6 | 0.06 | 0.3960 | 0.7420 |
| CALR | -0.05 | 0.4008 | 0.7420 |
| SERPINA7 | -0.05 | 0.4031 | 0.7420 |
| CFHR1 | 0.05 | 0.4086 | 0.7455 |
| IGK | 0.05 | 0.4216 | 0.7501 |
| APOH | 0.05 | 0.4242 | 0.7501 |
| FETUB | 0.05 | 0.4279 | 0.7501 |
| ADIPOQ | -0.05 | 0.4319 | 0.7501 |
| IGG1 | -0.05 | 0.4363 | 0.7501 |
| QSOX1 | 0.05 | 0.4383 | 0.7501 |
| IGF2 | -0.05 | 0.4389 | 0.7501 |
| SERPINF1 | -0.05 | 0.4472 | 0.7501 |
| APMAP | 0.05 | 0.4485 | 0.7501 |
| C1QC | -0.05 | 0.4489 | 0.7501 |
| JCHAIN | -0.05 | 0.4509 | 0.7501 |
| FGG | -0.05 | 0.4554 | 0.7501 |
| CP | 0.05 | 0.4598 | 0.7501 |
| C1S | 0.05 | 0.4619 | 0.7501 |
| ORM2 | 0.05 | 0.4652 | 0.7501 |
| MAN1A1 | -0.05 | 0.4709 | 0.7512 |
| CD44 | -0.05 | 0.4766 | 0.7512 |
| FBLN1 | -0.05 | 0.4767 | 0.7512 |
| PROCR | -0.05 | 0.4806 | 0.7516 |
| AGT | 0.04 | 0.4949 | 0.7647 |
| ANG | 0.04 | 0.5000 | 0.7647 |
| ATRN | 0.04 | 0.5002 | 0.7647 |
| GSN | 0.04 | 0.5037 | 0.7647 |
| VASN | -0.04 | 0.5104 | 0.7652 |
| FCGBP | -0.04 | 0.5167 | 0.7652 |
| APOC2 | 0.04 | 0.5212 | 0.7652 |
| LDHB | 0.04 | 0.5216 | 0.7652 |
| APOA1 | 0.04 | 0.5234 | 0.7652 |
| PROS1 | 0.04 | 0.5261 | 0.7652 |
| F7 | -0.04 | 0.5326 | 0.7693 |
| ICAM1 | -0.04 | 0.5378 | 0.7715 |
| ALDOB | 0.04 | 0.5716 | 0.8143 |
| GNPTG | -0.04 | 0.5778 | 0.8176 |
| SELL | 0.04 | 0.5854 | 0.8201 |
| GC | -0.04 | 0.5899 | 0.8201 |
| TFRC | 0.03 | 0.5914 | 0.8201 |
| SERPINA4 | 0.03 | 0.5970 | 0.8204 |
| LBP | 0.03 | 0.6033 | 0.8204 |
| APOC3 | 0.03 | 0.6080 | 0.8204 |
| PGLYRP2 | 0.03 | 0.6185 | 0.8204 |
| PTGDS | -0.03 | 0.6192 | 0.8204 |
| AZGP1 | 0.03 | 0.6277 | 0.8204 |
| PLTP | -0.03 | 0.6287 | 0.8204 |
| ORM1 | 0.03 | 0.6327 | 0.8204 |
| C2 | 0.03 | 0.6328 | 0.8204 |
| MASP1 | 0.03 | 0.6341 | 0.8204 |
| IGA2 | 0.03 | 0.6350 | 0.8204 |
| F12 | 0.03 | 0.6509 | 0.8277 |
| SERPIND1 | 0.03 | 0.6525 | 0.8277 |
| FCN3 | -0.03 | 0.6526 | 0.8277 |
| KLKB1 | 0.03 | 0.6713 | 0.8443 |
| CD14 | -0.03 | 0.6769 | 0.8443 |
| GPLD1 | 0.03 | 0.6779 | 0.8443 |
| HP | -0.03 | 0.6904 | 0.8548 |
| VWF | 0.03 | 0.6950 | 0.8554 |
| RNASE1 | -0.02 | 0.7085 | 0.8644 |
| C4BPA | 0.02 | 0.7106 | 0.8644 |
| CSF1R | 0.02 | 0.7176 | 0.8678 |
| RBP4 | 0.02 | 0.7249 | 0.8716 |
| C3 | 0.02 | 0.7355 | 0.8755 |
| AHSG | 0.02 | 0.7366 | 0.8755 |
| LPA | -0.02 | 0.7532 | 0.8852 |
| C4BPB | 0.02 | 0.7533 | 0.8852 |
| AMBP | 0.02 | 0.7647 | 0.8880 |
| APOA2 | 0.02 | 0.7654 | 0.8880 |
| MBL2 | 0.02 | 0.7718 | 0.8880 |
| SERPINF2 | 0.02 | 0.7727 | 0.8880 |
| GSTO1 | 0.02 | 0.7875 | 0.8974 |
| SERPINA3 | 0.02 | 0.7895 | 0.8974 |
| C1QB | -0.02 | 0.7990 | 0.9032 |
| LYVE1 | -0.02 | 0.8120 | 0.9099 |
| CRP | 0.02 | 0.8137 | 0.9099 |
| C4B | -0.01 | 0.8310 | 0.9155 |
| F13B | 0.01 | 0.8350 | 0.9155 |
| SH3BGRL3 | 0.01 | 0.8350 | 0.9155 |
| IGHG4 | 0.01 | 0.8413 | 0.9162 |
| SELENOP | 0.01 | 0.8511 | 0.9220 |
| MMRN1 | -0.01 | 0.8607 | 0.9276 |
| FGA | -0.01 | 0.8676 | 0.9300 |
| LRG1 | -0.01 | 0.8719 | 0.9300 |
| TIMP1 | -0.01 | 0.9287 | 0.9843 |
| C1QA | 0.01 | 0.9367 | 0.9843 |
| SAA1 | 0.00 | 0.9444 | 0.9843 |
| PROC | 0.00 | 0.9508 | 0.9843 |
| COL18A1 | 0.00 | 0.9545 | 0.9843 |
| F10 | 0.00 | 0.9574 | 0.9843 |
| LGALS3BP | 0.00 | 0.9598 | 0.9843 |
| IGHG3 | 0.00 | 0.9606 | 0.9843 |
| CETP | 0.00 | 0.9775 | 0.9956 |
| C9 | 0.00 | 0.9812 | 0.9956 |
| ITIH4 | 0.00 | 0.9913 | 0.9985 |
| SAA2 | 0.00 | 0.9967 | 0.9985 |
| CD163 | 0.00 | 0.9985 | 0.9985 |

Spearman correlation was used to determine correlations between continuous variables. The Benjamini and Hochberg’s correction was applied to calculate the FDR.

**Table S10. Correlation of serum miR-122 levels with serum proteins in all measurements ranked based on *P* values (*n* = 240 samples from *n* = 62 COVID-19 ICU patients).**

| **Protein** | **Spearman *r*** | ***P* value** | **FDR** |
| --- | --- | --- | --- |
| AFM | 0.29 | **<0.0001** | **0.0008** |
| ALDOB | 0.41 | **<0.0001** | **0.0008** |
| AMBP | 0.26 | **<0.0001** | **0.0008** |
| APOB | 0.33 | **<0.0001** | **0.0008** |
| APOC1 | 0.30 | **<0.0001** | **0.0008** |
| APOC2 | 0.31 | **<0.0001** | **0.0008** |
| APOC3 | 0.28 | **<0.0001** | **0.0008** |
| APOC4 | 0.32 | **<0.0001** | **0.0008** |
| APOE | 0.35 | **<0.0001** | **0.0008** |
| APOH | 0.29 | **<0.0001** | **0.0008** |
| BTD | 0.27 | **<0.0001** | **0.0008** |
| CPB2 | 0.33 | **<0.0001** | **0.0008** |
| CFI | 0.26 | **<0.0001** | **0.0008** |
| CLU | 0.31 | **<0.0001** | **0.0008** |
| C5 | 0.24 | **<0.0001** | **0.0008** |
| FN1 | 0.33 | **<0.0001** | **0.0008** |
| SERPINA4 | 0.35 | **<0.0001** | **0.0008** |
| MMRN1 | 0.25 | **<0.0001** | **0.0008** |
| GPLD1 | 0.24 | **<0.0001** | **0.0008** |
| PIGR | 0.28 | **<0.0001** | **0.0008** |
| PROZ | 0.30 | **<0.0001** | **0.0008** |
| PLXDC2 | 0.27 | **<0.0001** | **0.0008** |
| RBP4 | 0.35 | **<0.0001** | **0.0008** |
| SELENOP | 0.28 | **<0.0001** | **0.0008** |
| TTR | 0.41 | **<0.0001** | **0.0008** |
| AZGP1 | 0.34 | **<0.0001** | **0.0008** |
| CRP | -0.24 | **0.0002** | **0.0015** |
| CSF1R | 0.23 | **0.0003** | **0.0019** |
| F10 | 0.23 | **0.0003** | **0.0019** |
| IGM | 0.23 | **0.0003** | **0.0019** |
| SAA1 | -0.23 | **0.0003** | **0.0019** |
| TF | 0.23 | **0.0003** | **0.0019** |
| PLG | 0.23 | **0.0004** | **0.0024** |
| CFH | 0.22 | **0.0005** | **0.0028** |
| ITIH2 | 0.22 | **0.0005** | **0.0028** |
| PROC | 0.22 | **0.0005** | **0.0028** |
| C1RL | 0.22 | **0.0006** | **0.0033** |
| MBL2 | 0.21 | **0.0008** | **0.0042** |
| QSOX1 | 0.22 | **0.0008** | **0.0042** |
| COL18A1 | 0.21 | **0.0010** | **0.0051** |
| KNG1 | 0.21 | **0.0011** | **0.0054** |
| ITIH1 | 0.21 | **0.0013** | **0.0061** |
| SPARC | 0.21 | **0.0013** | **0.0061** |
| GP1BA | 0.21 | **0.0014** | **0.0065** |
| AGT | 0.20 | **0.0016** | **0.0072** |
| F2 | 0.20 | **0.0021** | **0.0093** |
| PPBP | 0.19 | **0.0030** | **0.0127** |
| SH3BGRL3 | 0.19 | **0.0030** | **0.0127** |
| IGFBP3 | 0.19 | **0.0033** | **0.0137** |
| FCGBP | 0.19 | **0.0040** | **0.0163** |
| APMAP | 0.18 | **0.0044** | **0.0176** |
| PROS1 | 0.18 | **0.0051** | **0.0200** |
| ICAM1 | 0.18 | **0.0053** | **0.0204** |
| ANG | 0.18 | **0.0057** | **0.0216** |
| CD44 | 0.18 | **0.0059** | **0.0219** |
| GP5 | 0.18 | **0.0060** | **0.0219** |
| PTGDS | 0.18 | **0.0063** | **0.0226** |
| VASN | 0.17 | **0.0068** | **0.024** |
| LCAT | 0.17 | **0.0071** | **0.0246** |
| IGHM | 0.17 | **0.0073** | **0.0249** |
| CFP | 0.17 | **0.0081** | **0.0272** |
| APOM | 0.17 | **0.0084** | **0.0273** |
| C6 | 0.17 | **0.0083** | **0.0273** |
| ITIH4 | 0.17 | **0.0095** | **0.0304** |
| SAA2 | -0.16 | **0.0107** | **0.0337** |
| PLTP | 0.16 | **0.0112** | **0.0348** |
| IGFALS | 0.16 | **0.0131** | **0.0395** |
| PRG4 | 0.16 | **0.0130** | **0.0395** |
| CETP | 0.16 | **0.0148** | **0.044** |
| A2M | -0.15 | **0.0178** | 0.0521 |
| KLKB1 | 0.15 | **0.0184** | 0.0532 |
| HRG | 0.15 | **0.0195** | 0.0556 |
| ECM1 | 0.15 | **0.0210** | 0.0590 |
| ORM1 | 0.15 | **0.0236** | 0.0646 |
| F13B | 0.15 | **0.0236** | 0.0646 |
| F12 | 0.14 | **0.0262** | 0.0708 |
| JCHAIN | 0.14 | **0.0272** | 0.0725 |
| IGK | 0.14 | **0.0314** | 0.0827 |
| CALR | 0.14 | **0.0331** | 0.0861 |
| APOA4 | 0.14 | **0.0340** | 0.0873 |
| LBP | -0.14 | **0.0367** | 0.0920 |
| LYVE1 | 0.14 | **0.0363** | 0.0920 |
| GNPTG | 0.13 | **0.0404** | 0.1000 |
| SAA4 | 0.13 | **0.0409** | 0.1001 |
| C1QA | 0.13 | **0.0438** | 0.1035 |
| C4BPB | 0.13 | **0.0433** | 0.1035 |
| SERPINA6 | 0.13 | **0.0428** | 0.1035 |
| MASP1 | 0.13 | **0.0450** | 0.1040 |
| SERPINF1 | 0.13 | **0.0450** | 0.1040 |
| C3 | 0.13 | **0.0456** | 0.1042 |
| CD5L | 0.13 | **0.0497** | 0.1124 |
| BCHE | 0.12 | 0.0548 | 0.1226 |
| SERPIND1 | 0.12 | 0.0601 | 0.1316 |
| TFRC | 0.12 | 0.0597 | 0.1316 |
| C8B | -0.12 | 0.0634 | 0.1372 |
| IGFBP6 | 0.12 | 0.0640 | 0.1372 |
| VWF | 0.12 | 0.0653 | 0.1386 |
| IGD | 0.12 | 0.0674 | 0.1416 |
| S100A9 | 0.12 | 0.0689 | 0.1433 |
| C4BPA | 0.12 | 0.0709 | 0.1452 |
| F11 | 0.12 | 0.0712 | 0.1452 |
| APCS | 0.12 | 0.0759 | 0.1533 |
| GSTO1 | 0.11 | 0.0768 | 0.1536 |
| F7 | 0.11 | 0.0830 | 0.1644 |
| AHSG | 0.11 | 0.0841 | 0.1650 |
| CD14 | -0.11 | 0.0851 | 0.1654 |
| HPX | -0.11 | 0.0884 | 0.1695 |
| LDHB | 0.11 | 0.0888 | 0.1695 |
| C7 | 0.11 | 0.0956 | 0.1808 |
| LRG1 | -0.11 | 0.1031 | 0.1932 |
| IGG1 | 0.10 | 0.1060 | 0.1969 |
| HABP2 | 0.10 | 0.1270 | 0.2317 |
| IGF2 | 0.10 | 0.1260 | 0.2317 |
| CP | 0.10 | 0.1282 | 0.2319 |
| TIMP1 | 0.10 | 0.1354 | 0.2428 |
| C1QB | 0.09 | 0.1471 | 0.2615 |
| S100A8 | 0.09 | 0.1490 | 0.2626 |
| SERPINF2 | 0.09 | 0.1519 | 0.2655 |
| CFD | 0.09 | 0.1600 | 0.2773 |
| HP | -0.09 | 0.1657 | 0.2830 |
| IGHG4 | 0.09 | 0.1660 | 0.2830 |
| GSN | 0.09 | 0.1695 | 0.2843 |
| SFTPB | -0.09 | 0.1694 | 0.2843 |
| SERPINC1 | 0.09 | 0.1742 | 0.2899 |
| A1BG | 0.09 | 0.1791 | 0.2957 |
| F5 | 0.09 | 0.1807 | 0.2959 |
| CST3 | 0.09 | 0.1852 | 0.3010 |
| FETUB | 0.08 | 0.2010 | 0.3241 |
| C8G | -0.08 | 0.2120 | 0.3392 |
| ATRN | 0.08 | 0.2161 | 0.3431 |
| APOF | 0.08 | 0.2195 | 0.3459 |
| APOA1 | 0.08 | 0.2269 | 0.3549 |
| CD163 | 0.07 | 0.2490 | 0.3846 |
| C1QC | 0.07 | 0.2533 | 0.3846 |
| SERPING1 | 0.07 | 0.2529 | 0.3846 |
| MASP2 | 0.07 | 0.2501 | 0.3846 |
| IGHG2 | 0.07 | 0.2873 | 0.4330 |
| CFHR1 | 0.07 | 0.2959 | 0.4428 |
| IGKC | 0.07 | 0.3015 | 0.4479 |
| CPN1 | -0.06 | 0.3199 | 0.4719 |
| SERPINA3 | -0.06 | 0.3643 | 0.5226 |
| ALB | 0.06 | 0.3637 | 0.5226 |
| LCN2 | 0.06 | 0.3601 | 0.5226 |
| VTN | -0.06 | 0.3602 | 0.5226 |
| APOL1 | 0.06 | 0.3892 | 0.5507 |
| GPX3 | 0.06 | 0.3942 | 0.5540 |
| F9 | 0.05 | 0.4049 | 0.5652 |
| IGHA1 | 0.05 | 0.4126 | 0.5697 |
| NID1 | 0.05 | 0.4136 | 0.5697 |
| GC | 0.05 | 0.4194 | 0.5739 |
| GLIPR2 | 0.05 | 0.4242 | 0.5767 |
| C2 | -0.05 | 0.4327 | 0.5844 |
| APOA2 | 0.05 | 0.4486 | 0.5996 |
| MPO | 0.05 | 0.4497 | 0.5996 |
| LPA | 0.05 | 0.4645 | 0.6039 |
| FCN3 | -0.05 | 0.4596 | 0.6039 |
| HBD | -0.05 | 0.4620 | 0.6039 |
| MMP9 | -0.05 | 0.4639 | 0.6039 |
| IGA2 | -0.05 | 0.4725 | 0.6104 |
| ITIH3 | 0.05 | 0.4756 | 0.6106 |
| PRSS3 | -0.05 | 0.4791 | 0.6114 |
| PCSK9 | 0.05 | 0.4836 | 0.6133 |
| PROCR | 0.04 | 0.4915 | 0.6159 |
| C1R | 0.04 | 0.5012 | 0.6242 |
| APOD | 0.04 | 0.5211 | 0.6354 |
| C9 | 0.04 | 0.5169 | 0.6354 |
| PGLYRP2 | 0.04 | 0.5224 | 0.6354 |
| PON1 | 0.04 | 0.5190 | 0.6354 |
| HBA1 | -0.04 | 0.5389 | 0.6517 |
| C8A | -0.04 | 0.5673 | 0.6817 |
| CPN2 | 0.04 | 0.5703 | 0.6817 |
| CHL1 | -0.04 | 0.5781 | 0.6871 |
| HPR | -0.03 | 0.5948 | 0.7029 |
| ORM2 | 0.03 | 0.6121 | 0.7193 |
| LGALS3BP | 0.03 | 0.6178 | 0.7219 |
| HBB | -0.03 | 0.6502 | 0.7540 |
| IGHG3 | 0.03 | 0.6525 | 0.7540 |
| CFB | 0.03 | 0.6705 | 0.7663 |
| RNASE1 | 0.03 | 0.6680 | 0.7663 |
| IGFBP4 | -0.03 | 0.6832 | 0.7765 |
| FBLN1 | -0.03 | 0.6882 | 0.7780 |
| CDH5 | -0.02 | 0.7070 | 0.7949 |
| C4B | -0.02 | 0.7465 | 0.8348 |
| CFHR5 | 0.02 | 0.7595 | 0.8448 |
| MAN1A1 | -0.02 | 0.7728 | 0.8550 |
| C4A | 0.02 | 0.7937 | 0.8685 |
| FGA | -0.02 | 0.7934 | 0.8685 |
| CLEC3B | 0.02 | 0.7975 | 0.8685 |
| SERPINA10 | 0.02 | 0.8024 | 0.8693 |
| TGFBI | -0.02 | 0.8096 | 0.8725 |
| SELL | -0.02 | 0.8145 | 0.8733 |
| DBH | -0.01 | 0.8255 | 0.8805 |
| SHBG | -0.01 | 0.8299 | 0.8807 |
| C1S | 0.01 | 0.8435 | 0.8906 |
| ADIPOQ | 0.01 | 0.8513 | 0.8943 |
| CTSD | 0.01 | 0.9105 | 0.9517 |
| B2M | -0.01 | 0.9257 | 0.9552 |
| SERPINA7 | -0.01 | 0.9276 | 0.9552 |
| THBS1 | -0.01 | 0.9276 | 0.9552 |
| IGFBP2 | 0.00 | 0.9421 | 0.9559 |
| LUM | 0.01 | 0.9382 | 0.9559 |
| PFN1 | 0.01 | 0.9376 | 0.9559 |
| FGG | 0.00 | 0.9534 | 0.9627 |
| SERPINA1 | 0.00 | 0.9899 | 0.9947 |
| FGB | 0.00 | 0.9965 | 0.9965 |

Spearman correlation was used to determine correlations between continuous variables. The Benjamini and Hochberg’s correction was applied to calculate the FDR.

**Table S11. COVID-19 severity classification (*n* = 45 non-severe vs *n* = 16 severe patients).**

| **Signature** | **Accur.** | **Sens.** | **Spec.** | **PPV** | **NPV** | **ROC AUC** |
| --- | --- | --- | --- | --- | --- | --- |
| **Singleton** |  |  |  |  |  |  |
| D-Dimer | 80.3 | 50.0 | 91.1 | 66.7 | 83.7 | 83.9 |
| miR-133a | 78.7 | 31.3 | 95.6 | 71.4 | 79.6 | 79.1 |
| miR-122 | 77.1 | 25.0 | 95.6 | 66.7 | 78.2 | 75.4 |
| RNAemia | 83.6 | 50.0 | 95.6 | 80.0 | 84.3 | 72.8 |
| BMI | 73.8 | 0.0 | 100.0 | 0.0 | 73.8 | 58.9 |
| Troponin | 72.1 | 0.0 | 97.8 | 0.0 | 73.3 | 58.7 |
| Age | 73.8 | 0.0 | 100.0 | 0.0 | 73.8 | 41.1 |
| **Binary** |  |  |  |  |  |  |
| D-Dimer + miR-122 | 85.2 | 68.8 | 91.1 | 73.3 | 89.1 | 94.4 |
| D-Dimer + miR-133a | 80.3 | 50.0 | 91.1 | 66.7 | 83.7 | 88.3 |
| miR-122 + miR-133a | 82.0 | 43.8 | 95.6 | 77.8 | 82.7 | 87.2 |
| Age + D-Dimer | 78.7 | 43.8 | 91.1 | 63.6 | 82.0 | 86.7 |
| D-Dimer + RNAemia | 78.7 | 50.0 | 88.9 | 61.5 | 83.3 | 85.6 |
| RNAemia + miR-133a | 86.9 | 68.8 | 93.3 | 78.6 | 89.4 | 85.4 |
| RNAemia + miR-122 | 85.3 | 62.5 | 93.3 | 76.9 | 87.5 | 81.3 |
| BMI + miR-133a | 78.7 | 37.5 | 93.3 | 66.7 | 80.8 | 80.2 |
| Troponin + RNAemia | 82.0 | 50.0 | 93.3 | 72.7 | 84.0 | 78.2 |
| Age + miR-122 | 77.1 | 25.0 | 95.6 | 66.7 | 78.2 | 76.5 |
| Age + miR-133a | 78.7 | 31.3 | 95.6 | 71.4 | 79.6 | 76.1 |
| Troponin + miR-133a | 77.0 | 31.3 | 93.3 | 62.5 | 79.2 | 75.2 |
| Age + RNAemia | 83.6 | 50.0 | 95.6 | 80.0 | 84.3 | 73.0 |
| Troponin + miR-122 | 77.0 | 25.0 | 95.6 | 66.7 | 78.2 | 70.2 |
| BMI + RNAemia | 83.6 | 50.0 | 95.6 | 80.0 | 84.3 | 69.8 |
| BMI + miR-122 | 75.4 | 18.8 | 95.6 | 60.0 | 76.8 | 69.4 |
| Age + BMI | 72.1 | 0.0 | 97.8 | 0.0 | 73.3 | 57.6 |
| Age + Troponin | 72.1 | 0.0 | 97.8 | 0.0 | 73.3 | 42.0 |
| **Triplet** |  |  |  |  |  |  |
| D-Dimer + miR-122 + RNAemia | 86.9 | 68.8 | 93.3 | 78.6 | 89.4 | 94.1 |
| D-Dimer + miR-122 + miR-133a | 83.6 | 68.8 | 88.9 | 68.8 | 88.9 | 94.1 |
| D-Dimer + miR-122 + Age | 85.2 | 68.8 | 91.1 | 73.3 | 89.1 | 93.7 |
| RNAemia + miR-122 + miR-133a | 85.3 | 68.8 | 91.1 | 73.3 | 89.1 | 89.8 |
| D-Dimer + Age + miR-133a | 82.0 | 56.3 | 91.1 | 69.2 | 85.4 | 89.6 |
| Age + miR-122 + miR-133a | 80.3 | 43.8 | 93.3 | 70.0 | 82.4 | 88.5 |
| D-Dimer + RNAemia + miR-133a | 78.7 | 56.3 | 86.7 | 60.0 | 84.8 | 88.2 |
| BMI + miR-122 + miR-133a | 82.0 | 43.8 | 95.6 | 77.8 | 82.7 | 85.7 |
| Troponin + miR-122 + miR-133a | 82.0 | 43.8 | 95.6 | 77.8 | 82.7 | 85.2 |
| Age + miR-122 + RNAemia | 85.3 | 62.5 | 93.3 | 76.9 | 87.5 | 83.5 |
| BMI + RNAemia + miR-133a | 80.3 | 56.3 | 88.9 | 64.3 | 85.1 | 83.1 |
| Age + miR-133a + RNAemia | 86.9 | 68.8 | 93.3 | 78.6 | 89.4 | 82.4 |
| Troponin + RNAemia + miR-122 | 85.2 | 62.5 | 93.3 | 76.9 | 87.5 | 80.6 |
| Troponin + RNAemia + miR-133a | 85.2 | 68.8 | 91.1 | 73.3 | 89.1 | 78.7 |
| BMI + miR-122 + RNAemia | 85.2 | 62.5 | 93.3 | 76.9 | 87.5 | 78.7 |
| BMI + Age + miR-133a | 78.7 | 37.5 | 93.3 | 66.7 | 80.8 | 78.0 |
| Troponin + miR-122 + Age | 77.0 | 25.0 | 95.6 | 66.7 | 78.2 | 73.5 |
| Troponin + Age + miR-133a | 77.0 | 31.3 | 93.3 | 62.5 | 79.2 | 73.3 |
| BMI + miR-122 + Age | 77.0 | 25.0 | 95.6 | 66.7 | 78.2 | 69.8 |

Results of singleton, binary and triplet signatures are ranked from high to low ROC AUC. Abbreviations: Accur: accuracy (%), NPV: negative predictive value (%), PPV: positive predictive value (%), ROC AUC: area under the receiver operating characteristic curve (%), Sens: sensitivity (%), Spec: specificity (%).

**Table S12. COVID-19 ICU outcome prediction (*n* = 48 survivors vs *n* = 17 non-survivors).**

| **Signature** | **Accur.** | **Sens.** | **Spec.** | **PPV** | **NPV** | **ROC AUC** |
| --- | --- | --- | --- | --- | --- | --- |
| **Singleton** |  |  |  |  |  |  |
| Age | 78.5 | 35.3 | 93.8 | 66.7 | 80.4 | 75.6 |
| RNAemia | 80.0 | 58.8 | 87.5 | 62.5 | 85.7 | 73.9 |
| Troponin | 72.3 | 5.9 | 95.8 | 33.3 | 74.2 | 70.3 |
| miR-122 | 72.3 | 5.9 | 95.8 | 33.3 | 74.2 | 67.5 |
| D-Dimer | 72.3 | 0.0 | 97.9 | 0.0 | 73.4 | 66.1 |
| miR-133a | 73.7 | 11.2 | 97.1 | 59.5 | 74.4 | 64.2 |
| BMI | 73.8 | 0.0 | 100 | 0.0 | 73.8 | 61.7 |
| **Binary** |  |  |  |  |  |  |
| Age + RNAemia | 83.1 | 52.9 | 93.8 | 75.0 | 84.9 | 85.1 |
| D-Dimer + RNAemia | 78.5 | 58.8 | 85.4 | 58.8 | 85.4 | 82.4 |
| Age + miR-133a | 81.5 | 52.9 | 91.7 | 69.2 | 84.6 | 81.6 |
| Age + D-Dimer | 78.5 | 41.2 | 91.7 | 63.6 | 81.5 | 78.9 |
| RNAemia + miR-122 | 75.4 | 41.2 | 87.5 | 53.9 | 80.8 | 78.6 |
| Troponin + RNAemia | 75.4 | 47.1 | 85.4 | 53.3 | 82.0 | 77.9 |
| Age + Troponin | 78.5 | 41.2 | 91.7 | 63.6 | 81.5 | 77.5 |
| RNAemia + miR-133a | 76.9 | 52.9 | 85.4 | 56.3 | 83.7 | 77.3 |
| Age + BMI | 80.0 | 47.1 | 91.7 | 66.7 | 83.0 | 77.2 |
| Troponin + miR-133a | 73.8 | 23.5 | 91.7 | 50.0 | 77.2 | 74.1 |
| BMI + RNAemia | 78.5 | 52.9 | 87.5 | 60.0 | 84.0 | 73.7 |
| Age + miR-122 | 75.4 | 35.3 | 89.6 | 54.6 | 79.6 | 71.4 |
| BMI + miR-133a | 73.8 | 11.8 | 95.8 | 50.0 | 75.4 | 71.1 |
| Troponin + miR-122 | 72.3 | 5.9 | 95.8 | 33.3 | 74.2 | 69.9 |
| D-Dimer + miR-133a | 70.8 | 11.8 | 91.7 | 33.3 | 74.6 | 69.7 |
| miR-122 + miR-133a | 73.9 | 11.8 | 95.8 | 50.0 | 75.4 | 68.9 |
| D-Dimer + miR-122 | 70.8 | 0.0 | 95.8 | 0.0 | 73.0 | 64.5 |
| BMI + miR-122 | 75.4 | 5.9 | 100 | 100 | 75.0 | 63.0 |
| **Triplet** |  |  |  |  |  |  |
| Age + miR-122 + RNAemia | 83.1 | 52.9 | 93.8 | 75.0 | 84.9 | 88.6 |
| Age + miR-133a + RNAemia | 78.5 | 47.1 | 89.6 | 61.5 | 82.7 | 84.3 |
| BMI + Age + miR-133a | 78.5 | 41.2 | 91.7 | 63.6 | 81.5 | 82.8 |
| Troponin + RNAemia + miR-133a | 78.5 | 52.9 | 87.5 | 60.0 | 84.0 | 81.4 |
| D-Dimer + RNAemia + miR-133a | 80.0 | 58.8 | 87.5 | 62.5 | 85.7 | 80.8 |
| D-Dimer + Age + miR-133a | 76.9 | 35.3 | 91.7 | 60.0 | 80.0 | 80.7 |
| Age + miR-122 + miR-133a | 78.5 | 35.3 | 93.8 | 66.7 | 80.4 | 79.8 |
| Troponin + Age + miR-133a | 76.9 | 41.2 | 89.6 | 58.3 | 81.1 | 79.8 |
| Troponin + miR-122 + Age | 78.5 | 41.2 | 91.7 | 63.6 | 81.5 | 78.4 |
| D-Dimer + miR-122 + Age | 78.5 | 41.2 | 91.7 | 63.6 | 81.5 | 78.3 |
| BMI + miR-122 + Age | 81.5 | 52.9 | 91.7 | 69.2 | 84.6 | 77.3 |
| D-Dimer + miR-122 + RNAemia | 75.4 | 52.9 | 83.3 | 52.9 | 83.3 | 75.7 |
| RNAemia + miR-122 + miR-133a | 76.9 | 41.2 | 89.6 | 58.3 | 81.1 | 74.1 |
| BMI + RNAemia + miR-133a | 80.0 | 58.8 | 87.5 | 62.5 | 85.7 | 72.7 |
| Troponin + miR-122 + miR-133a | 73.8 | 23.5 | 91.7 | 50.0 | 77.2 | 72.5 |
| Troponin + miR-122 + RNAemia | 75.4 | 52.9 | 83.3 | 52.9 | 83.3 | 71.0 |
| BMI + miR-122 + miR-133a | 73.8 | 17.6 | 93.8 | 50.0 | 76.3 | 69.2 |
| BMI + miR-122 + RNAemia | 80.0 | 52.9 | 89.6 | 64.3 | 84.3 | 68.2 |
| D-Dimer + miR-122 + miR-133a | 70.8 | 17.6 | 89.6 | 37.5 | 75.4 | 65.7 |

Results of singleton, binary and triplet signatures are ranked from high to low ROC AUC. Abbreviations: Accur: accuracy (%), NPV: negative predictive value (%), PPV: positive predictive value (%), ROC AUC: area under the receiver operating characteristic curve (%), Sens: sensitivity (%), Spec: specificity (%).

**Supplementary References**

1. Brandenburger T, Lorenzen JM. Diagnostic and Therapeutic Potential of microRNAs in Acute Kidney Injury. *Front Pharmacol* Frontiers; 2020;**11**:657.

2. Willeit P, Skroblin P, Kiechl S, Fernández-Hernando C, Mayr M. Liver microRNAs: potential mediators and biomarkers for metabolic and cardiovascular disease? *Eur Heart J* 2016;**37**:3260–3266.

3. Sunderland N, Skroblin P, Barwari T, Huntley RP, Lu R, Joshi A, Lovering RC, Mayr M. MicroRNA Biomarkers and Platelet Reactivity. *Circ Res* 2017;**120**:418–435.

4. Zampetaki A, Kiechl S, Drozdov I, Willeit P, Mayr U, Prokopi M, Mayr A, Weger S, Oberhollenzer F, Bonora E, Shah A, Willeit J, Mayr M. Plasma MicroRNA Profiling Reveals Loss of Endothelial MiR-126 and Other MicroRNAs in Type 2 Diabetes. *Circ Res* 2010;**107**:810–817.

5. Willeit P, Zampetaki A, Dudek K, Kaudewitz D, King A, Kirkby NS, Crosby-Nwaobi R, Prokopi M, Drozdov I, Langley SR, Sivaprasad S, Markus HS, Mitchell JA, Warner TD, Kiechl S, Mayr M. Circulating MicroRNAs as Novel Biomarkers for Platelet Activation. *Circ Res* 2013;**112**:595–600.

6. Kaudewitz D, Skroblin P, Bender LH, Barwari T, Willeit P, Pechlaner R, Sunderland NP, Willeit K, Morton AC, Armstrong PC, Chan M V., Lu R, Yin X, Gracio F, Dudek K, Langley SR, Zampetaki A, Rinaldis E de, Ye S, Warner TD, Saxena A, Kiechl S, Storey RF, Mayr M. Association of MicroRNAs and YRNAs With Platelet Function. *Circ Res* 2016;**118**:420–432.

7. Giza DE, Fuentes-Mattei E, Bullock MD, Tudor S, Goblirsch MJ, Fabbri M, Lupu F, Yeung S-CJ, Vasilescu C, Calin GA. Cellular and viral microRNAs in sepsis: mechanisms of action and clinical applications. *Cell Death Differ* Nature Publishing Group; 2016;**23**:1906–1918.

8. Braza-Boïls A, Barwari T, Gutmann C, Thomas MR, Judge HM, Joshi A, Pechlaner R, Shankar-Hari M, Ajjan RA, Sabroe I, Storey RF, Mayr M. Circulating MicroRNA Levels Indicate Platelet and Leukocyte Activation in Endotoxemia Despite Platelet P2Y12 Inhibition. *Int J Mol Sci* 2020;**21**:2897.

9. Tacke F, Roderburg C, Benz F, Cardenas DV, Luedde M, Hippe H-J, Frey N, Vucur M, Gautheron J, Koch A, Trautwein C, Luedde T. Levels of Circulating miR-133a Are Elevated in Sepsis and Predict Mortality in Critically Ill Patients. *Crit Care Med* 2014;**42**:1096–1104.

10. Wu S-C, Yang JC-S, Rau C-S, Chen Y-C, Lu T-H, Lin M-W, Tzeng S-L, Wu Y-C, Wu C-J, Hsieh C-H. Profiling circulating microRNA expression in experimental sepsis using cecal ligation and puncture. *PLoS One* 2013;**8**:e77936.

11. Chen L, Xie W, Wang L, Zhang X, Liu E, Kou Q. MiRNA-133a aggravates inflammatory responses in sepsis by targeting SIRT1. *Int Immunopharmacol* 2020;**88**:106848.

12. Donaldson A, Natanek SA, Lewis A, Man WD-C, Hopkinson NS, Polkey MI, Kemp PR. Increased skeletal muscle-specific microRNA in the blood of patients with COPD. *Thorax* 2013;**68**:1140–1149.

13. Schulte C, Barwari T, Joshi A, Theofilatos K, Zampetaki A, Barallobre-Barreiro J, Singh B, Sörensen NA, Neumann JT, Zeller T, Westermann D, Blankenberg S, Marber M, Liebetrau C, Mayr M. Comparative Analysis of Circulating Noncoding RNAs Versus Protein Biomarkers in the Detection of Myocardial Injury. *Circ Res* 2019;**125**:328–340.

14. Navickas R, Gal D, Laucevičius A, Taparauskaitė A, Zdanytė M, Holvoet P. Identifying circulating microRNAs as biomarkers of cardiovascular disease: a systematic review. *Cardiovasc Res* 2016;**111**:322–337.

15. Jaguszewski M, Osipova J, Ghadri J-R, Napp LC, Widera C, Franke J, Fijalkowski M, Nowak R, Fijalkowska M, Volkmann I, Katus HA, Wollert KC, Bauersachs J, Erne P, Lüscher TF, Thum T, Templin C. A signature of circulating microRNAs differentiates takotsubo cardiomyopathy from acute myocardial infarction. *Eur Heart J* 2014;**35**:999–1006.

16. Wang F, Long G, Zhao C, Li H, Chaugai S, Wang Y, Chen C, Wang D. Plasma microRNA-133a is a new marker for both acute myocardial infarction and underlying coronary artery stenosis. *J Transl Med* BioMed Central; 2013;**11**:222.

17. Pattarayan D, Thimmulappa RK, Ravikumar V, Rajasekaran S. Diagnostic Potential of Extracellular MicroRNA in Respiratory Diseases. *Clin Rev Allergy Immunol* 2018;**54**:480–492.

18. Ferruelo A, Peñuelas Ó, Lorente JA. MicroRNAs as biomarkers of acute lung injury. *Ann Transl Med* 2018;**6**:34.

19. Gilje P, Gidlöf O, Rundgren M, Cronberg T, Al-Mashat M, Olde B, Friberg H, Erlinge D. The brain-enriched microRNA miR-124 in plasma predicts neurological outcome after cardiac arrest. *Crit Care* BioMed Central; 2014;**18**:R40.
